# Serpin-driven green camouflage and NIR fluorescence in frogs

**DOI:** 10.1126/sciadv.aeg5510

**Published:** 2026-09-16

**Authors:** Ravichandran Vignesh, Tri Vu, Grace Harvey, Luca Menozzi, Jesse Delia, William White, Pohan Lin, Erini Galatis, Sönke Johnsen, Junjie Yao, Carlos Taboada

**Affiliations:** ^1^ Division of Biology and Biological Engineering, California Institute of Technology, Pasadena, CA, USA.; ^2^ Stephenson School of Biomedical Engineering, University of Oklahoma, Norman, OK, USA.; ^3^ Department of Biomedical Engineering, Duke University, Durham, NC, USA.; ^4^ American Museum of Natural History, New York City, NY, USA.; ^5^ Biology Department, Vassar College, Poughkeepsie, NY, USA.; ^6^ Department of Biology, Duke University, Durham, NC, USA.

## Abstract

Biliverdin is one of the few endogenous green pigments in animals, yet its use in camouflage and fluorescence is limited by rapid degradation or excretion. Multiple arboreal frogs have overcome this constraint by evolving biliverdin-binding serpins (BBSs)—proteins that stabilize biliverdin. Here, we show that BBSs have convergently evolved high-affinity biliverdin binding comparable to hormone-receptor interactions and tune biliverdin’s spectral properties to produce leaf-like green coloration. We further show that BBSs from independent lineages exhibit distinct spectral properties, including near-infrared fluorescence. Unlike most animal serpins, which are rapidly cleared following proteolytic cleavage, a glassfrog’s BBS is naturally cleaved while retaining full biliverdin-binding affinity, revealing an unusual decoupling between serpin proteolysis and ligand binding. Using photoacoustic tomography, we map the distribution of BBS throughout the body, uncovering a mechanism of protein-based camouflage. These findings provide insights into serpin evolution and protein-based coloration while establishing a foundation for amphibian-inspired near-infrared molecular probe design.

## INTRODUCTION

In ectothermic vertebrates, coloration is primarily produced by skin chromatophores (*1*, *2*). However, multiple arboreal frog lineages have evolved an alternative mechanism characterized by a strong reduction in the number of melanin-containing chromatophores and the high accumulation of the bile pigment biliverdin throughout their tissues—including blood, lymph, muscle, skin, and bones (*3*, *4*). This accumulation of biliverdin, a condition known as chlorosis, modulates light absorption and transmission resulting in leaf-like green camouflage (*4*).

Biliverdin is the first breakdown product of heme from senescent red blood cells and exhibits green coloration due to absorption in the violet (Soret band) and red (Q band) regions of the spectrum (*5*). Although intrinsically green, biliverdin’s absorption spectrum can be strongly influenced by its conformation, protonation state, and interactions with its molecular environment (*5*, *6*). Leaf-dwelling frogs in the families Hylidae and Centrolenidae have independently co-opted a liver-derived serpin, termed the biliverdin-binding serpin (BBS), which binds biliverdin and exploits its spectral tunability (*4*, *7*). By altering biliverdin’s conformation and electronic structure, BBSs shift both the position and magnitude of its absorption peaks toward wavelengths that approximate those of plant chlorophyll, a structurally related tetrapyrrole (*4*, *7*). These spectral changes include a red shift of the Soret band and a more structured, blue-shifted Q band that exhibits a threefold increase in absorption (*4*). Across frog lineages, amino acid substitutions in BBSs produced distinct absorption profiles that shaped frog coloration, resulting in optical phenotypes ranging from bluish to light green (*4*, *7*).

The use of biliverdin to create colors in vertebrates presents physiological challenges due to its rapid enzymatic reduction to the yellow pigment bilirubin by the enzyme biliverdin reductase (BLVRA) (*1*, *8*), as well as its rapid clearance from systemic circulation even under extreme hemolysis or following direct biliverdin administration (*9*, *10*). In the absence of specific binding or sequestration mechanisms, free biliverdin is therefore short lived in circulation. This physiological constraint has biological consequences, as biliverdin is one of the few green pigments in animals, yet its use to modulate colors in nature is rare (*11*, *12*). Preserving high concentrations of biliverdin in vivo thus requires mechanisms to minimize its chemical degradation and to slow its natural clearance (*13*, *14*). The widespread use of BBSs among leaf-dwelling frogs of different lineages suggests that BBSs have convergently evolved mechanisms to bind biliverdin with high affinity.

Biliverdin binding in proteins is typically associated with a rigidification of biliverdin, which results in fluorescence emission, primarily in the red (630 to 700 nm) and near-infrared (NIR; >700 nm) regions of the spectrum (*15*–*17*). This photochemical behavior has important biological and practical implications. The NIR region corresponds to a wavelength range in which absorption and scattering by biological tissues are minimal, often referred to as the optical window for tissue imaging (*18*). This suggests that frogs expressing BBSs could emit NIR fluorescence detectable through overlying tissues, a characteristic shared with chlorophyll-associated photophysical systems in plants (*19*), potentially expanding the repertoire of optical traits in amphibians and contributing to leaf-associated camouflage. Whether BBSs produce detectable fluorescence in vivo remains unknown, as quenchers present in frog tissues under normal physiological conditions could attenuate BBS fluorescence.

Understanding the biophysical properties that enable biliverdin binding and spectral tuning in BBSs will shed light on the evolution of protein-based coloration and provide a framework for linking the molecular evolution of a single protein to a leaf-like phenotype. In addition, their fluorescence in the NIR positions BBSs as a promising template in developing bioinspired optical probes that permit deep tissue penetration. Last, identifying BBS variants that have been evolutionarily tuned to enhance biliverdin binding may reveal molecular strategies for overcoming the rapid turnover of biliverdin in vivo—a key limitation of NIR fluorescent probes that rely on endogenous biliverdin (*13*, *17*, *20*).

Here, we describe a new BBS (TpBBS) from the glassfrog *Teratohyla pulverata* (Centrolenidae) and characterize its biophysical and optical properties. We show that TpBBS is a hyperstable protein that binds biliverdin with high affinity, approaching that of hormone-carrier and antibody-epitope interactions (*21*, *22*). Extending our analysis to two additional independent origins of BBSs in treefrogs (*Boana punctata* and *Sphaenorhynchus lacteus*), we demonstrate that distinct BBSs adopt different conformational states and produce species-specific green coloration. Using spectroscopy, NIR fluorescence imaging, and hyperspectral photoacoustic tomography (PAT), we further show that BBSs preserve their optical properties in vivo, substantially extending the known spectral range of amphibian fluorescence.

## RESULTS

### BBSs and green leaf-color camouflage

**Fig. 1.**
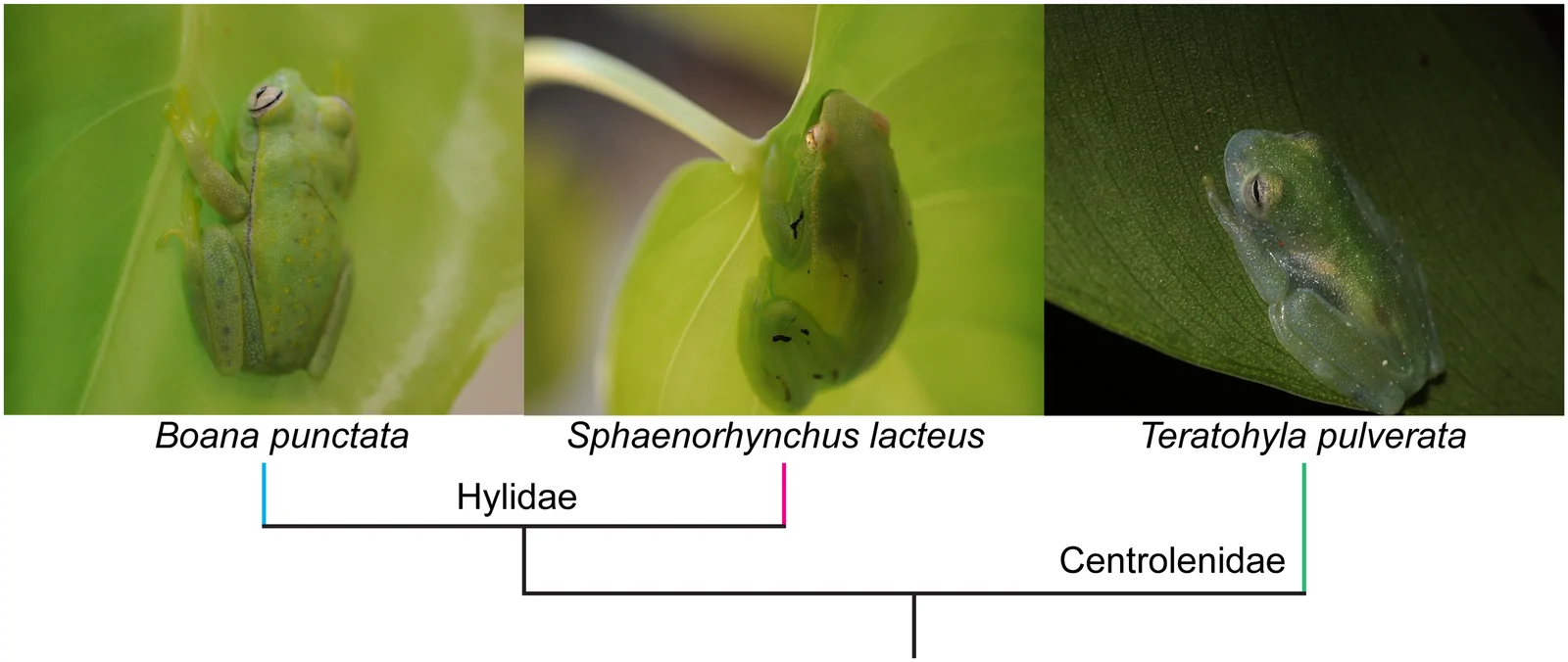
Chlorotic arboreal frogs of the Hylidae and Centrolenidae family. The polka-dot treefrog *B. punctata* from Suriname (Hylidae: Cophomantini), the hatchet-faced treefrog *S. lacteus* from Suriname (Hylidae: Sphaenorhynchini), and the powdered glassfrog *T. pulverata* (Centrolenidae) from Nicaragua. These three species represent three independent evolutionary origins of high BBS expression, tissue transparency, and biological mirrors coating one or more organs or parietal peritonea (*4*).

**Fig. 2.**
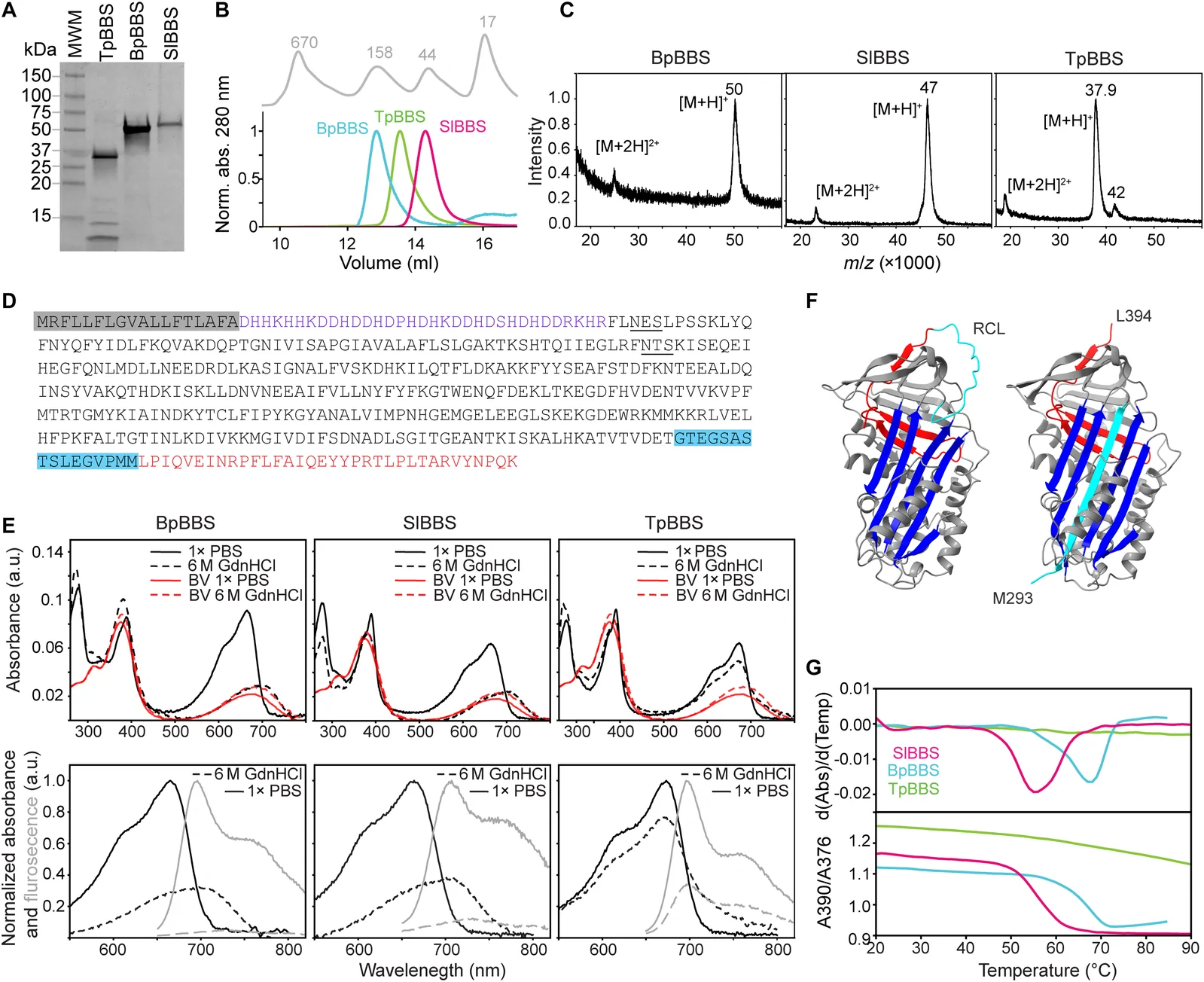
Purification and characterization of BBSs from chlorotic frogs. (**A**) Coomassie brilliant blue (CBB)–stained SDS-PAGE of purified BBS from the tissues of *B. punctata*, *S. lacteus*, and *T. pulverata.* (**B**) Size exclusion chromatogram of purified endogenous BpBBS (cyan), TpBBS (green), SlBBS (pink), and standards (gray). (**C**) MALDI-TOF of the different BBSs. (**D**) Amino acid sequence of TpBBS identified from de novo transcriptomics and proteomics; signal peptide (gray), N terminus (purple), reactive center loop (RCL; cyan), and C terminus (red), with potential glycosylation sites underlined. (**E**) Top*:* Absorbance spectra for Bp-, Sl-, and TpBBSs in 1× PBS and denaturing conditions (6 M GdnHCl) compared to free biliverdin (BV). Bottom: Normalized Q band absorbance (black) and fluorescence (gray) of BBSs in 1× PBS (solid lines) and in 6 M GdnHCl (dotted line), respectively. The fluorescence spectrum represented here was collected by exciting the samples at 630 nm. (**F**) AlphaFold predicted structure of TpBBS—uncleaved stressed conformation (left) and hyperstable relaxed conformation, with the RCL (cyan) inserted into β sheet A (blue) of the serpin fold (right). (**G**) Melting curves of different BBS (absorbance versus *T*) representing the loss of structural fold and release of biliverdin (bottom) and the first derivative (dA/dT versus *T*) (top). *m/z*, mass/charge ratio.

The arboreal frogs *T. pulverata*, *B. punctata*, and *S. lacteus* examined in this study (Fig. 1) represent three independent evolutionary origins of BBSs (*4*). Their translucent skin reveals large accumulations of subcutaneous blue lymph. To investigate the role of BBSs in producing plant-like green coloration, we purified BBSs from each species, TpBBS, BpBBS, and SlBBS (Fig. 2A), and characterized their biophysical and optical properties.

Size exclusion analysis of the purified endogenous proteins revealed a symmetric peak for all the BBSs, with the predicted hydrodynamic radii ordered as BpBBS>TpBBS>SlBBS (Fig. 2B). The molecular weights of the purified BBSs were estimated by matrix-assisted laser desorption/ionization–time-of-flight (MALDI-TOF) to be 37.9 kDa for TpBBS, 47 kDa for SlBBS, and 50 kDa for BpBBS (Fig. 2, B and C).

The sequences of BpBBS and SlBBS have been previously reported (*4*). We determined the full sequence of TpBBS, using tandem mass spectrometry (MS/MS), by searching the resulting peptides against the de novo liver transcriptome of *T. pulverata* (Fig. 2C). The predicted molecular weight of TpBBS is 46.5 kDa, and the sequence includes two N-glycosylation consensus motifs (Fig. 2D). Multiple sequence alignment of TpBBS, SlBBS, BpBBS, and other serpins shows that TpBBS shares 76 to 83% sequence identity with BBSs from other glassfrogs (Centrolenidae) but only 46 to 52% identity with those from treefrogs of the family Hylidae (fig. S1).

Absorbance and fluorescence spectra for all BBSs are shown in Fig. 2E. The Soret band exhibited maximal absorbance at 389 to 392 nm across all BBSs, red-shifted relative to free biliverdin in 1× phosphate-buffered saline (PBS) (Fig. 2E). The Q band, in contrast, varied in both spectral shape and peak position; TpBBS showed a maximum at 673 to 676 nm, whereas BpBBS and SlBBS peaked at 666 nm (Fig. 2E). Upon excitation, either at 390 or 630 nm, all BBSs showed a broad fluorescence emission with a peak at 695 to 705 nm, extending into the NIR region of the spectrum (Fig. 2E and fig. S2).

BpBBS is known to contain two N-glycosylation sites (*4*), whereas SlBBS has a single glycosylation consensus sequence. To assess glycosylation as a posttranslational modification in the different BBSs, we treated the samples with peptide *N*-glycosidase F (PNGase F). BpBBS and SlBBS were efficiently deglycosylated upon PNGase F treatment (fig. S3). In contrast, the molecular weight of TpBBS remained unchanged after PNGase F treatment (fig. S3), suggesting that it is not glycosylated, carries glycans of low molecular weight, or contains glycans resistant to PNGase F cleavage.

### TpBBS is a thermally stable BBS in the relaxed conformation

Most serpins are serine protease inhibitors that regulate protease activity in diverse physiological processes using a unique mechanism centered on a structural feature called the reactive center loop (RCL) (*23*). Inhibition occurs when a protease cleaves the RCL, which drives the conformational change causing the insertion of RCL into the central β sheet A of the serpin fold (Fig. 2F) (*24*). This marked structural transition is possible because the native fold is metastable, or stressed, with the exposed RCL stable under physiological conditions, yet energetically far from the protein’s optimal state. After RCL is cleaved and inserted into the serpin fold, it adopts a relaxed conformation that enhances serpin stability, reflected in an increased melting temperature (*T*_m_) (*25*, *26*). While the empirical molecular weights of BpBBS and SlBBS matched their theoretical predictions, TpBBS appeared smaller in both SDS–polyacrylamide gel electrophoresis (SDS-PAGE) and MALDI-TOF, suggesting that in *T. pulverata*, TpBBS might be naturally cleaved. Bottom-up mass spectrometry of the purified TpBBS gel band at ∼37 kDa (Fig. 2A) revealed ∼80% sequence coverage (Fig. 2C) and did not include any peptides from either the N- or C-terminal regions (highlighted in purple and red in Fig. 2C), suggesting that TpBBS is truncated at both ends. To determine whether the cleaved fragment is associated with the protein in the native state, we crosslinked TpBBS (fig. S3B) and analyzed the sample with MS/MS after trypsinization. The crosslinked sample contained the C-terminal peptide (fig. S3C), bringing the coverage to 91% (fig. S3D) but showed no evidence of the missing N-terminal region, indicating that this portion is either absent or extensively modified. This was further confirmed by mass spectrometry analysis of the native TpBBS, which revealed an additional peak at 4088 mass/charge ratio (fig. S3E). Analysis of the fragmentation patterns of this peptide indicated that TpBBS is naturally cleaved at M393 (fig. S3, C to E) in its native state, within the RCL (fig. S1A), which suggests that the protein might adopt a hyperstable relaxed conformation, with the RCL inserted into β sheet A of the serpin fold (Fig. 2F). To test this hypothesis—and to compare it with BpBBS and SlBBS, which are presumably in the native stressed conformation—we incubated all three proteins in 6 M guanidine hydrochloride (GdnHCl) for 1 hour. In BpBBS and SlBBS, biliverdin rapidly dissociated from the apoprotein upon denaturation, as evidenced by a two- to threefold decrease in Q band absorbance (Fig. 2E), a hypsochromic shift of the Soret band from 390 to 376 nm, and a pronounced reduction in NIR fluorescence emission (Fig. 2E). In contrast, TpBBS exhibited only minor changes in absorbance and NIR fluorescence upon GdnHCl treatment, indicating a highly stable fold typical of serpins in the relaxed conformation (Fig. 2F) (*25*, *26*).

To further assess whether the endogenous TpBBS is in relaxed conformation, we performed a thermal unfolding of all the BBSs and calculated their melting temperature (*T*_m_). The melting curves of BBS (A390/A376 versus *T*) and the first derivative (dA/dT versus *T*) are shown in Fig. 2G. *T*_m_ values for BpBBS and SlBBS were calculated to be 67° and 56°C, respectively, whereas TpBBS showed no temperature-dependent change in the ratio of absorbance. These results indicate that TpBBS is highly stable, consistent with the behavior observed under GdnHCl denaturation. This exceptional stability supports the notion that TpBBS exists in the thermodynamically more stable relaxed conformation, with the cleaved RCL inserted into β sheet A (Fig. 2F).

### BBSs bind biliverdin with high affinity

**Fig. 3.**
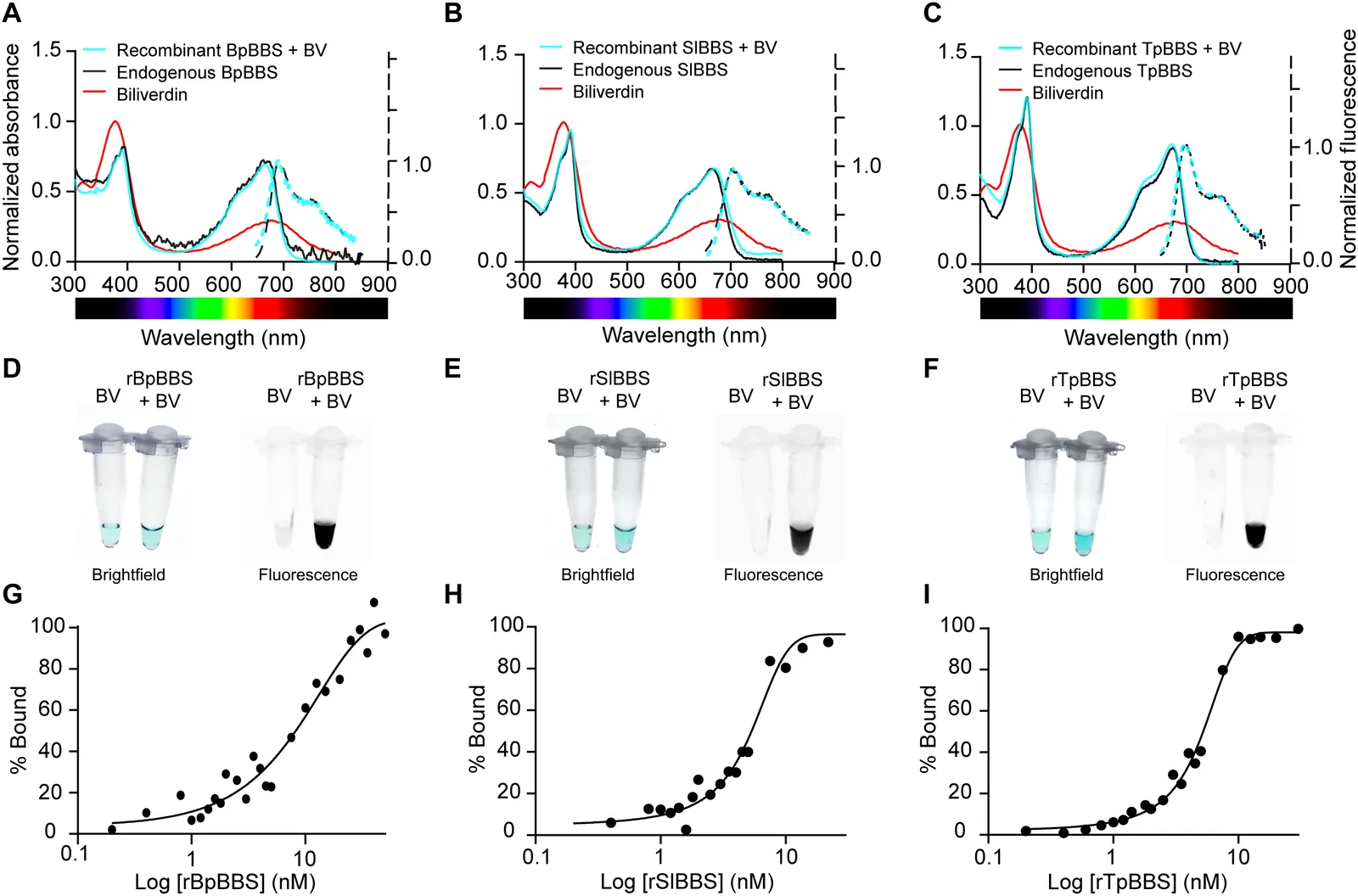
Biliverdin binding in the recombinant BBS. (**A** to **C**) Normalized absorbance [normalized to the Soret band of free biliverdin (BV) at the same concentration] and fluorescence (dotted lines, excitation at 630 nm for BpBBS and 390 nm for SlBBS and TpBBS, emission at 650 to 850 nm) of biliverdin-bound recombinant BBSs (cyan) compared to endogenous BBSs (black) and free biliverdin (red). (**D** to **F**) Bright-field and fluorescence pictures of free biliverdin and recombinant biliverdin-bound BBSs. The color of biliverdin changes from green to blue/bluish-green, and samples become fluorescent. Fluorescence images were acquired using a Bio-Rad ChemidocMP Imager with a Cy5.5 filter. Fluorescence of the samples is shown in black. (**G** to **I**) Ligand binding curves (% bound versus concentration) of recombinant BBSs after 4 hours of equilibration.

In frogs, chlorosis refers to a physiological state associated with unusually high concentrations of biliverdin (*4*), a condition that contrasts with most vertebrates, where biliverdin is rapidly excreted or enzymatically reduced to bilirubin (*8*, *10*). This suggests that their BBSs have high affinity for biliverdin, thereby extending its lifetime in vivo by sequestering it and preventing its excretion (*3*, *4*). To evaluate the affinity of BBSs for biliverdin, we expressed recombinant BBSs in *Escherichia coli* and performed fluorescence titration experiments in the NIR range. To verify whether the recombinant proteins retained binding properties comparable to their endogenous counterparts, we recorded absorbance and fluorescence spectra for both sets of proteins (Fig. 3, A to C). The spectra were indistinguishable in both the Soret and Q bands, in terms of amplitude and peak position, indicating identical optical properties. The extinction coefficients of the Soret and Q band were determined for all three biliverdin-bound BBSs and revealed a 2.5 to 3.5 times increase in absorption in the Q band relative to free biliverdin (Table 1 and fig. S4). The change in optical properties induced by the binding of biliverdin to BBSs is observed as an increase in the color saturation of the solution and the emergence of fluorescence emission upon excitation in the far-red region (Fig. 3, D to F). The quantum yield of the proteins upon excitation at 650 nm was 1.39% for BpBBS, 0.58% for SlBBS, and 1.24% for TpBBS.

**Table 1.** Optical and ligand binding properties of recombinant BBSs.

Sample	ε_280_ (M^−1^ cm^−1^)	ε_Soret_ (M^−1^ cm^−1^)	ε_Q-band_ (M^−1^ cm^−1^)	*K*_d_ (nM)	Quantum yield
BpBBS	28,880	32,072	28,034	7.5	1.39%
SlBBS	35,870	38,393	26,229	5.0	0.58%
TpBBS	33,350	52,809	36,449	5.0	1.24%

To quantify the interaction between BBS and biliverdin, we determined the apparent dissociation constant (*K*_d_) by maintaining biliverdin at a fixed concentration (the limiting reagent) while titrating increasing amounts of recombinant apoprotein. We repeated the experiments at different biliverdin concentrations and equilibration times (fig. S5) to ensure that measurements were conducted in the binding rather than titration regime (*27*). At biliverdin concentration as low as 15 nM, the apparent *K*_d_ values were 5 nM for TpBBS, 5 nM for SlBBS, and 7.5 nM for BpBBS—still within the titration regime [estimated *K*_d_ ≈ ½ (limiting reagent)] (Fig. 3, G to I). Further testing at lower protein concentrations was limited by the sensitivity of the fluorescence assay.

### TpBBS has a high affinity for biliverdin in the stressed and relaxed states

While the protease recognition sites and inhibitory roles of specific serpins have been extensively characterized in humans and mice (*24*, *25*, *28*), information on amphibian serpins remains limited (*4*, *24*, *29*). Given that endogenous TpBBS is cleaved in its relaxed serpin conformation—a structural state that, in other serpins such as the hormone carrier CBG and others, has been associated with a drastic decrease in ligand affinity (*21*, *30*)—we decided to investigate whether the relaxation observed in vivo affects biliverdin binding.

**Fig. 4.**
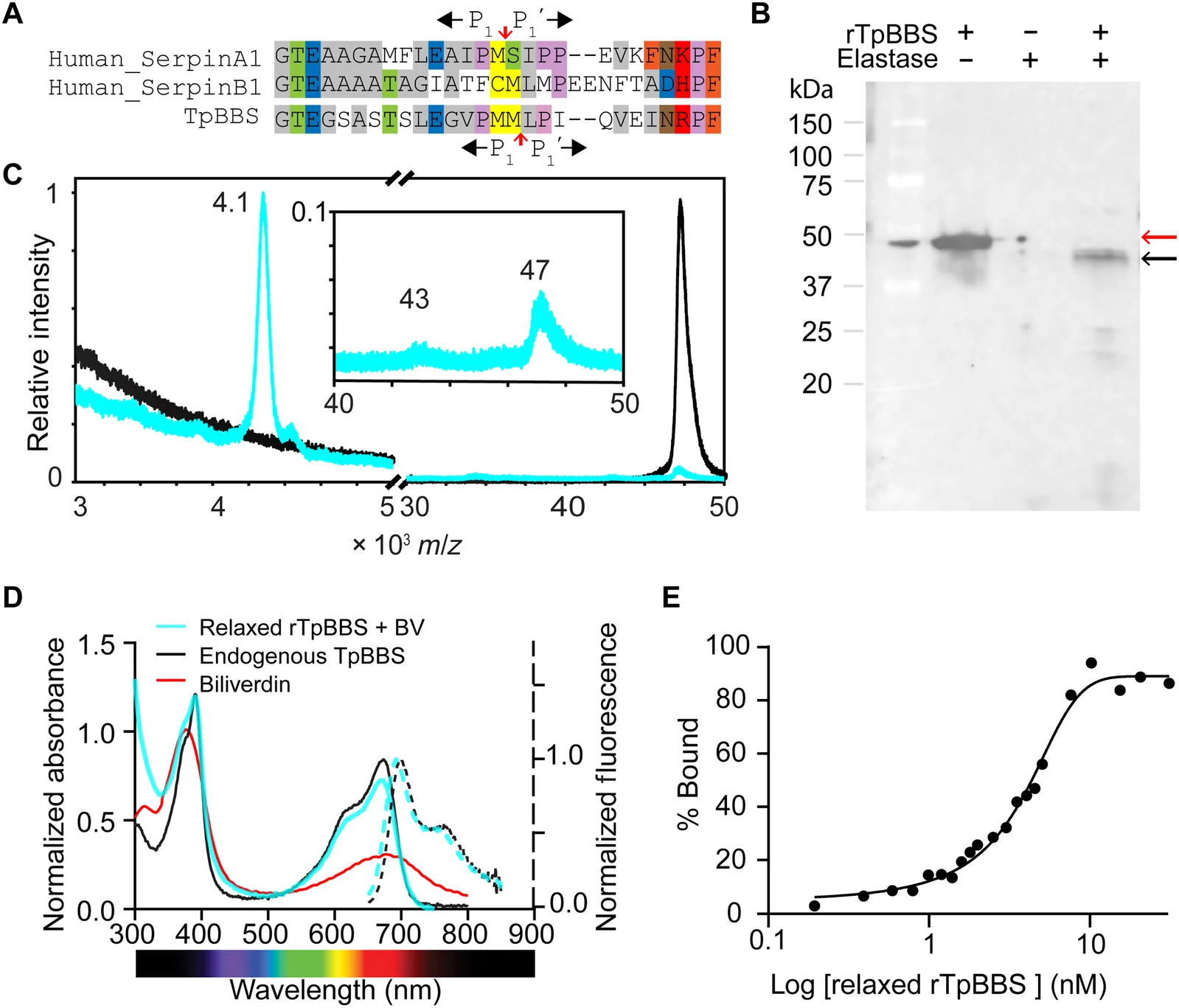
Relaxed rTpBBS binds biliverdin with high affinity. (**A**) Sequence alignment of RCL sequences of known neutrophil elastase inhibitors and TpBBS. The scissile bond of TpBBS is shifted one amino acid relative to the other canonical inhibitors (red arrow). (**B**) Cleavage of rTpBBS by (human) neutrophil elastase monitored with Western blotting using anti-his antibody. The cleaved rTpBBS (black arrow) exhibited lower molecular weight than the uncleaved protein (red arrow). (**C**) Normalized MALDI-TOF of the relaxed rTpBBS (blue) compared to untreated rTpBBS (black). The peak at ∼4.1 kDa confirms the cleavage of rTpBBS at the scissile bond, in the same position as the endogenous TpBBS. Inset shows the cleaved protein (43 kDa) as analyzed by MALDI-TOF. (**D**) Absorbance spectra of relaxed rTpBBS bound to biliverdin (blue) normalized to the Soret band of free biliverdin at the same concentration and fluorescence (dotted lines, excitation at 390 nm and emission at 650 to 850 nm), endogenous TpBBS (black), and free biliverdin (red). (**E**) Ligand binding curves (% bound versus concentration) of relaxed rTpBBS after 4 hours of equilibration.

To identify a protease capable of cleaving the TpBBS RCL for further biochemical tests, we aligned its sequence with those of human serpins (Fig. 4A). RCL of TpBBS shares the highest sequence identity with human SerpinA1 (α_1_-antitrypsin), a well-characterized inhibitor of neutrophil elastase (*23*). Hence, we predicted the presence of a human neutrophil elastase cleavage site in these BBSs (Fig. 4A). We therefore incubated recombinant TpBBS (rTpBBS) with human neutrophile elastase and observed effective cleavage, resulting in a reduction in apparent molecular weight, consistent with the loss of the C-terminal portion following cleavage at the scissile bond (Fig. 4B). Using MALDI-TOF, we confirmed that the released peptide is 4088 Da (Fig. 4C and fig. S6), consistent with the C-terminal fragment observed in the endogenous protein (fig. S3E). These results indicate that the scissile bond in TpBBS is shifted by one position toward the C terminus relative to other elastase inhibitors, such as SerpinA1 and Serpin B1 (Fig. 4A). Titration of the relaxed rTpBBS with biliverdin resulted in spectral features (absorbance and fluorescence) matching the biliverdin-bound endogenous TpBBS (Fig. 4D). The determined apparent *K*_d_ was 3.5 nM (Fig. 4E), which suggests that the high affinity for biliverdin is not affected by the proteolytic cleavage of TpBBS.

### BBSs in vivo: Skin, bones, lymph, and ova

**Fig. 5.**
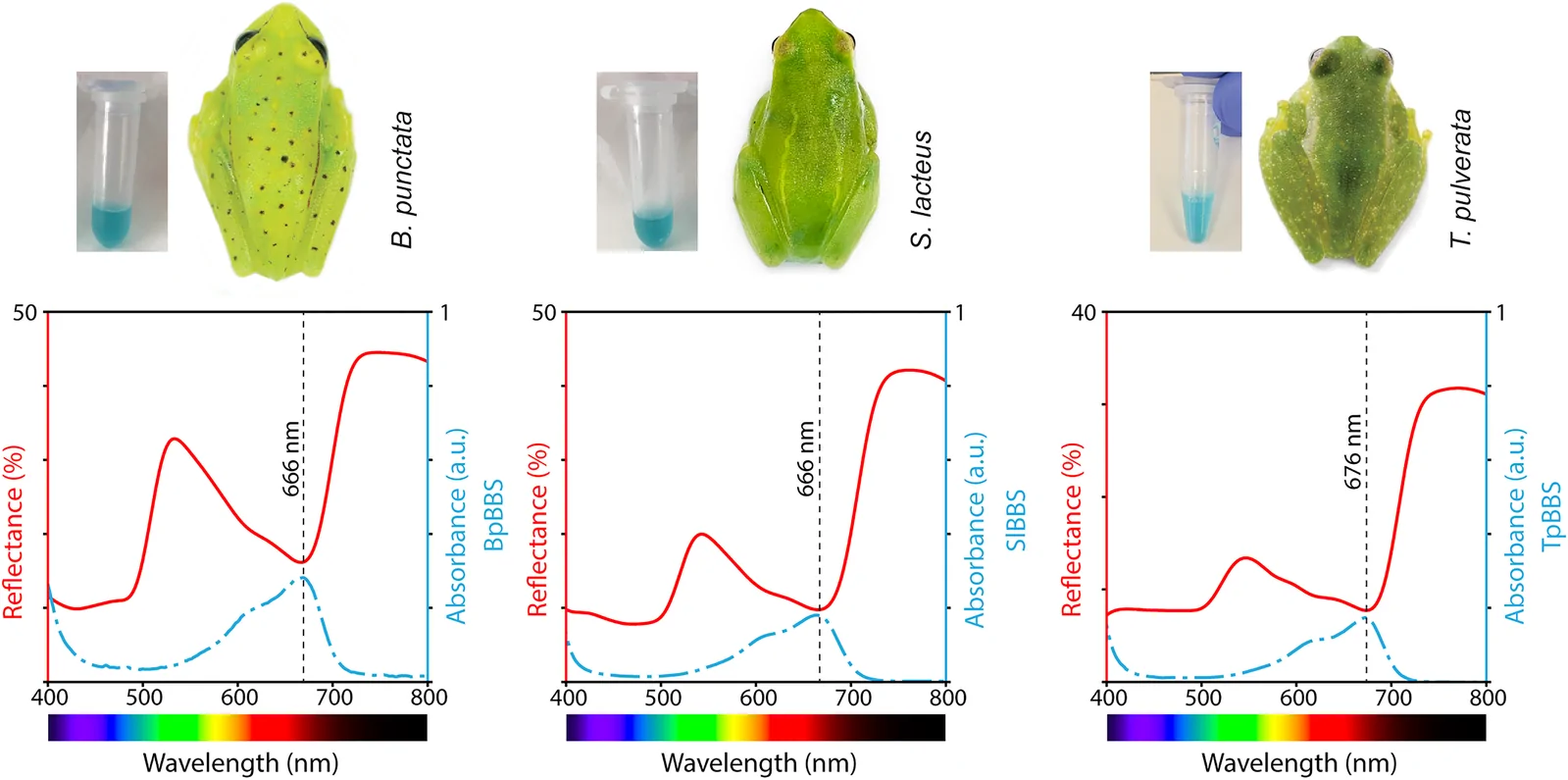
Whole-body reflectance and BBS absorbance in two treefrogs and a glassfrog. Reflectance spectra of (red, solid line) from left to right: *B. punctata*, *S. lacteus*, and *T. pulverata* (left to right) show a peak in the green portion of the spectrum, a local minimum in the far-red, and a steep increase of reflectance in the red-NIR boundary, known as red edge that characterizes living, photosynthesizing vegetation. The absorbance spectrum collected from the extracted proteins is represented in dashed-dotted blue lines. The BBS used for absorbance measurements is shown next to the frogs. The peak and shape of BBSs’ absorbance perfectly mirror the valley in reflectance explaining the sharp reduction in the intensity of the radiated light in the red region (see also fig. S7).

To evaluate whether the purified BBSs described here in vitro can reproduce the hues and saturation observed in in vivo, and to test whether the purified BBSs exhibit the same optical properties in vivo, we measured the reflectance spectra of live frogs (Fig. 5). Reflectance quantifies the spectrally resolved fraction of backscattered light following its interaction with tissue components, including absorption by intra- and extracellular pigments. The frogs’ reflectance spectra show a peak at 520 to 550 nm and a steep increase at the far-red/NIR boundary, characteristic of the red edge in living vegetation (fig. S7) (*31*, *32*). Reflectance is markedly lower in the blue portion of the spectrum—likely due to absorption by carotenoids and pteridines (*4*, *7*)—and in the red region (660 to 680 nm) (Fig. 5 and fig. S7).

Analysis of these reflectance spectra reveals that the reduced reflectance in the red region mirrors the absorbance of pure BBSs, consistently matching their absorbance peaks and spectral features, including a blue-shifted absorbance shoulder (Fig. 5). These results suggest that the reflectance spectra of frogs are strongly modulated by the absorbance properties of BBSs.

**Fig. 6.**
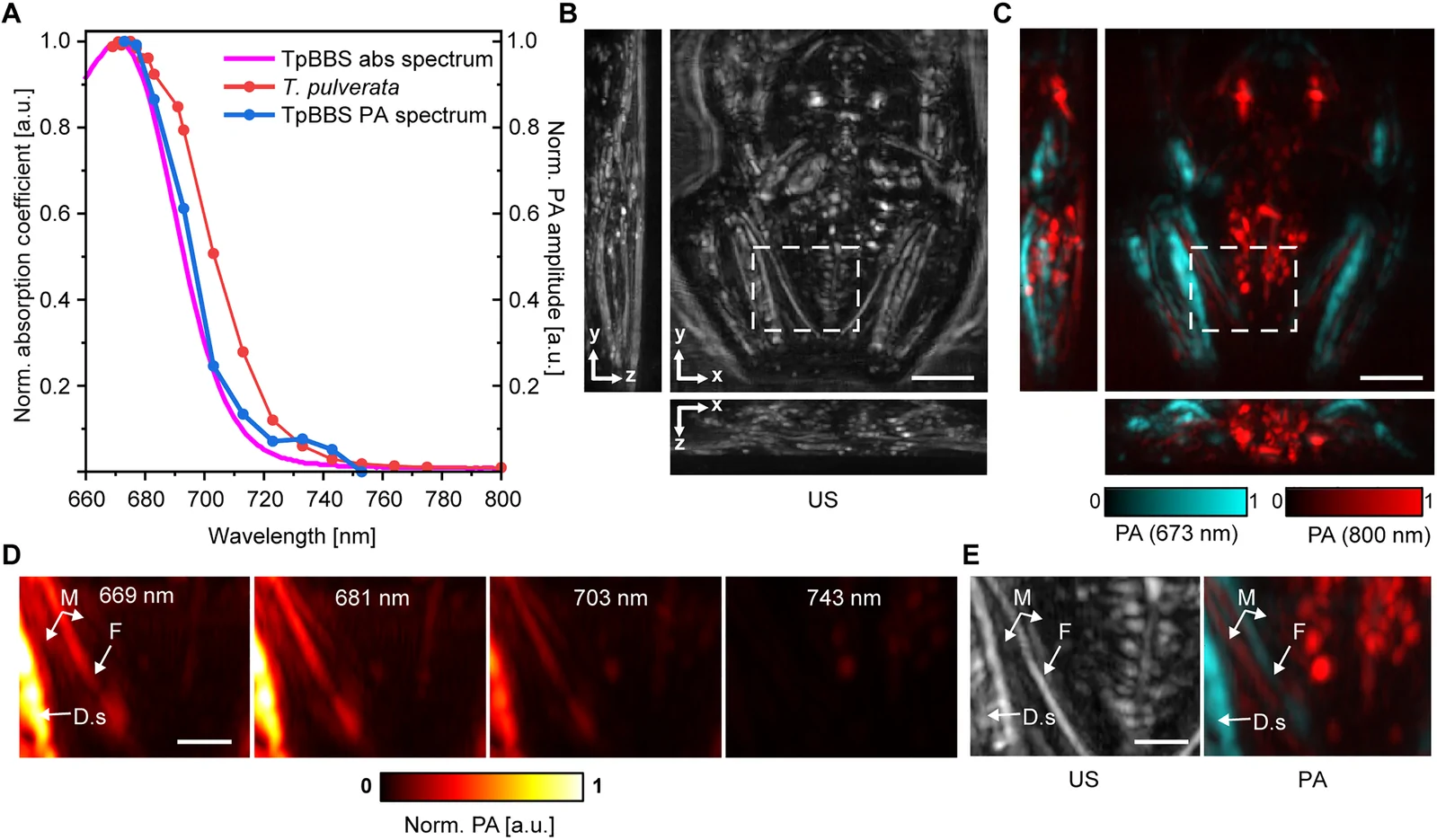
Hyperspectral PAT of *T. pulverata* reveals the presence of TpBBS in subcutaneous tissues. (**A**) Average PA amplitude of live frog (blue) and TpBBS-filled tube (pink) compared with the TpBBS’ optical absorption spectrum (red). (**B**) Referenced ultrasound (US) image of a sleeping frog, projected along three different dimensions, and (**C**) corresponding overlay of PA images showing TpBBS with 673-nm PA signal (light blue) and other pigments such as hemoglobin and melanin at 800 nm (red). (**D** and **E**) Zoomed-in region of the US and PA images at increasing wavelengths (D) and US and PA at 673 and 800 nm, respectively (E). TpBBS is distributed across multiple tissues, with high concentrations in the bones [femur (F)], lymph, dorsal skin (D.s.), and muscles (M). Because the internal organs of glassfrogs are covered by broadband reflectors that backscatter most of the incident light, the light fluence is attenuated in the dorsal skin immediately overlying the internal organs, which results in weak PA signals. Scale bars, 5 mm (B and C) and 2 mm (D and E).

BBSs generate whole-body green coloration through their widespread distribution, including in blood plasma, lymph, and other extracellular fluids (*3*, *7*, *9*). In many chlorotic frog species, bones exhibit blue-green hues, and in females, oocytes also display green coloration (*4*, *33*–*35*). To further investigate the distribution of BBSs in these tissues in vivo, including within highly pigmented tissues such as blood, we implemented a PAT approach. We first verified that the photoacoustic (PA) spectrum of purified TpBBS matches its absorption spectrum (Fig. 6A and fig. S8). Next, we imaged an individual *T. pulverata* frog to analyze the spectra properties of internal tissues (Fig. 6B). The PA spectra obtained in vivo (Fig. 6, A and C) revealed that TpBBS is present in multiple tissues and can be clearly distinguished from other pigments, such as hemoglobin and melanin. Our results indicate that TpBBS is highly concentrated in bones, lymph, muscles, internal organs, and blood within vessels, reaching values higher than 200 μM in the skin and lymph.

### BBSs produce NIR fluorescence in frogs

**Fig. 7.**
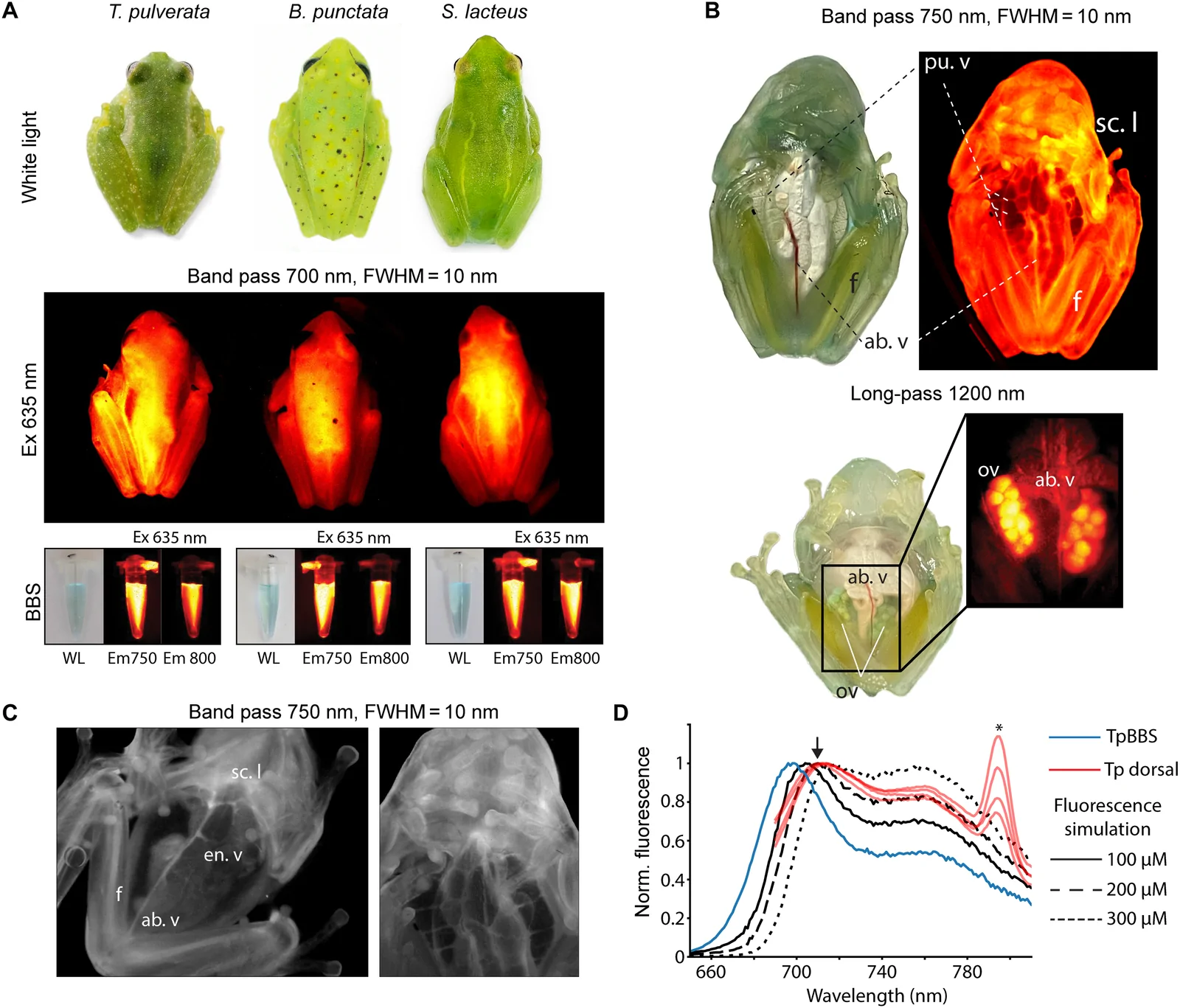
BBSs cause chlorotic hylids and glassfrogs to fluoresce in the NIR when excited in the red/far-red spectral range. Fluorescence emission can be observed in various body regions, including the dorsal (**A**) and the ventral side of the body (**B** and **C**), in both sleeping and active frogs (see also movie S1 and fig. S10), and in purified BBS samples. NIR images can be visualized either using pseudocolor palettes (A and B) or grayscale (C). BBSs are concentrated in subcutaneous lymph, blood plasma, bones, and ova. NIR can be detected from inside the body using band-pass (FWHM = 10 nm) and long-pass filters, revealing the distribution of BBS within the organism, and it can be observed at wavelengths as high as 1200 nm. Excitation is at 635 nm (FWHM = 2 nm) for (A) and 660 nm (FWHM = 20 nm) for (B) and (C), respectively. ab. v., abdominal vein; en. v., enteric vein; f*.*, femur; ova., ovaries; pu. v., pulmonary veins; sc. l., subcutaneous lymph. (**D**) The NIR emission peak is modulated in vivo due to fluorescence reabsorption. With increasing concentrations of BBS in skin and lymph emission peak shifts from 697 to ∼710 nm (arrow), compared to pure TpBBS. Asterisk “*” indicates the Raman peak.

It has been suggested that fluorescence emission may play a role in visual signaling or camouflage in frogs (*33*, *36*–*38*). However, many strong fluorophores extracted from amphibian skin have been shown to be quenched and barely detectable in vivo (*36*). To test whether the fluorescence properties of BBSs are preserved in vivo, we imaged multiple individuals using a series of band-pass and long-pass emission filters spanning the first and second NIR [short-wavelength infrared (SWIR)] windows (Fig. 7, A to C; fig. S9; and movie S1).

In many fluorescent vertebrates, fluorescence is predominantly confined to the ultraviolet (UV) or visible wavelengths, and the distribution of fluorophores is restricted to certain tissues, often within chromatophore cells (*2*, *33*, *39*). Contrastingly, NIR emission from these frogs can be detected broadly across tissues, including the skin, blood, lymph, bones, and ovaries, extending to wavelengths beyond 1200 nm in the SWIR window (Fig. 7C). Notably, emission is amplified in regions underlain by broadband reflective biological structures, such as the peritoneum and the fasciae surrounding muscles and internal organs. To confirm that the emission in vivo exhibits the same properties as those of isolated BBSs in vitro, we obtained fluorescence spectra of adult individuals. Different emission spectra collected from *T. pulverata* are red-shifted compared to those of pure TpBBS (Fig. 7D and fig. S10), due to light reabsorption, which typically occurs in samples with high concentrations of fluorophores. To assess the effect of BBS concentration on the reabsorption of the emission spectra, we performed a Monte Carlo simulation of the fluorescence emission using a three-layered model that includes an outer skin layer, a middle lymph layer of variable concentrations, and a lower layer composed of a broad-band ideal reflector. An increase in the concentration of BBS in the lymph can effectively modulate the emission, shifting the peak to 710 nm or more, closely matching the spectra obtained in vivo (Fig. 7D). These results confirm that BBSs are responsible for the fluorescence emission of the frogs.

## DISCUSSION

### BBSs and the creation of leaf-like colors

In this work, we characterized the biophysical and optical properties of three BBSs from arboreal frogs from the Americas (Hylids and Centrolenids) and confirmed that they can explain the organisms’ leaf-like green coloration and their fluorescence emission in the NIR window. Our results show that these BBSs are spectrally red-shifted relative to free biliverdin (in the Soret band) and absorb substantially more red light—a feature that generates deep green saturation in a manner analogous to chlorophyll in plants. By increasing the absorption coefficient of the Q band 2.5- to 3.5-fold and introducing a blue shift in its spectral absorption, chlorotic frogs generate a more saturated green hue than could be achieved with free biliverdin at similar or even higher concentrations.

Frogs that do not rely on biliverdin for coloration instead depend on dermal chromatophore units composed of multiple pigmentary cell types in the skin. Among these, melanin-containing melanophores play a key role by broadly absorbing light and selectively removing red and NIR wavelengths from backscattered light (*1*, *2*). By contrast, glassfrogs and treefrogs that use BBSs, together with a reduction in skin melanin, efficiently subtract red wavelengths while allowing NIR light to be reflected and transmitted with minimal attenuation (*4*, *40*–*43*). This produces an optical phenotype characterized by a sharp increase in reflected light at the transition between the red and NIR regions, known as the red edge, a feature shared with chlorophyll-bearing plants (*4*, *7*, *44*–*46*).

In the present work, we demonstrated that the spectral features of the Q band of BBSs are detectable in whole animal reflectance spectra. Using hyperspectral PAT, we further showed that the absorption spectral signatures of deep tissues—including bones, vessels, and lymph—closely match those of BBSs. These results reinforce our conclusion that amphibian coloration arises from a combination of physiological, anatomical, and biochemical mechanisms that integrate heterogeneous systems such as blood metabolism, lymphatic circulation, and bone morphogenesis to produce desired optical traits. The use of biliverdin to generate optical traits in animals requires that the pigment either be stably preserved in tissues for extended periods (*3*, *9*, *47*) or produced at rates exceeding its excretion or enzymatic conversion of biliverdin to bilirubin (*10*).

In nonchlorotic frogs, high doses of biliverdin injected directly into the lymphatic sacs—or artificially elevated biliverdin levels through hemolysis—failed to reproduce the chlorotic phenotype, as the animals rapidly excreted the excess pigment (*10*). This observation suggests that elevated biliverdin synthesis alone is insufficient to maintain high concentrations in vivo and that additional mechanisms are required for the accumulation of the pigment. In this work, using fluorescence titration experiments performed across multiple concentrations and equilibration times revealed that BBSs bind biliverdin with dissociation constants in the low nanomolar range and potentially tighter. These affinities are comparable to those of hormone-carrying serpins such as CBG (*21*) and are three to four orders of magnitude higher than those of other vertebrate biliverdin-binding proteins (*48*). The biliverdin binding in BBSs is also three to five orders of magnitude stronger than that of an alkaloid-binding serpin from poison dart frogs (*K*_d_ ∼ 30 to 50 μM) (*49*). Such tight binding likely accounts for the exceptionally high biliverdin concentrations observed in chlorotic frogs. The fact that nonchlorotic frogs cannot accumulate large concentrations of biliverdin might be explained by the lack of an ortholog or any other globulin with sufficient affinity for biliverdin or a reduced expression of their BBS homolog.

Additional biochemical pathways also contribute to interspecific differences in biliverdin accumulation. The inability of most frogs to accumulate high concentrations of biliverdin may therefore not solely reflect the absence of a high-affinity carrier protein but also differences in biliverdin metabolism, transport, excretion, or the expression and catalytic efficiency of BLVRA (*8*–*10*, *50*). Nevertheless, the reported Michaelis constant (*K*_m_) values of vertebrate BLVRAs (0.3 to 7 μM) (*51*–*54*) indicate biliverdin interactions approximately 100 to 1000 times weaker than those of BBSs, suggesting that the exceptionally tight binding of BBSs is likely a key factor permitting the retention and accumulation of biliverdin in chlorotic frogs.

Only one prior study has reported dissociation constants for recombinant BpBBS, estimating a *K*_d_ of ∼2.7 μM (*29*). This discrepancy likely arises from differences in expression systems, titration conditions [e.g., measurements performed in the titration versus binding regime (*27*)], or buffer compositions that could influence binding. Notably, the previously reported absorbance spectrum of recombinant BpBBS differs substantially from that of the endogenous protein (*4*, *29*), exhibiting an 18-nm shift in the Q band and a 4-nm shift in the Soret band, suggesting a different conformation of biliverdin in the binding pocket (*5*, *6*, *55*). In contrast, our current results show that the spectral features of recombinant and endogenous BBSs closely align, suggesting that the biliverdin-binding properties measured in vitro here accurately reflect those in vivo. These findings also raise the possibility of multiple biliverdin conformations or alternative binding mechanisms.

### Thermal stability and relaxed conformation in vivo

We found that SlBBS and BpBBS have high thermal stability with melting points above 55°C, which aligns with the temperatures reported for other serpins in nature (*24*, *26*). In this work, we purified and sequenced the BBS from the glassfrog *T. pulverata* and found that TpBBS adopts a hyperstable, relaxed conformation, with a cleaved RCL likely inserted in the serpin fold. The relaxed conformation accounts for the high thermal stability of TpBBS, even at temperatures as high as 95°C. Now, there is no clear interpretation or biological role for this increase in stability within the species’ natural history. Higher thermal stability reflects greater thermodynamic stability and is often associated with increased resistance to unfolding, aggregation, and polymerization, as proteins typically require partial unfolding before self-association (*56*, *57*). Higher thermal stability suggests that the protein can retain its native conformation and function despite prolonged exposure to temperature fluctuations, thereby reducing the likelihood of thermal denaturation (*58*). Additional experiments will be required to determine whether the increased thermal stability observed in BBSs is simply a biophysical consequence of the canonical serpin transition from the stressed to the relaxed state, or whether this conformational change itself has adaptive value by enhancing protein stability under physiological conditions.

Furthermore, cleaved serpins are rarely detected in vivo, either because they are produced at low levels or are rapidly cleared after inhibiting their target proteases (*59*, *60*). However, their levels can increase in inflammatory cases (*61*), coagulation disorders (*62*), or tissue injury (*63*). While the enzyme responsible for cleaving TpBBS in vivo is unknown, our sequence alignments suggest that neutrophil elastase may be one of the serine proteases that cleave the RCL. While the biochemical information about amphibian elastases is limited to an ortholog in *Xenopus tropicalis* (*64*), its catalytic specificity closely matches that of other vertebrates. Our experimental results showed that human neutrophil elastase cleaves the rTpBBS at M393, consistent with the cleavage site detected in endogenous proteins. This cleavage site is shifted one amino acid toward the C terminus of the protein relative to the canonical position of the scissile bond in other serpins (*23*). These results offer an opportunity to investigate structural, biochemical, and evolutionary correlations associated with the length and specificity of the RCL in serpins. It is still unclear whether TpBBS acts as an inhibitor or an alternative protease substrate with purely noninhibitory functions in vivo.

Despite undergoing cleavage, TpBBS showed no detectable differences in absorbance between the stressed and relaxed forms, suggesting that serpin cleavage does not alter the ligand binding or the optical effect on the animals. In contrast, corticosteroid- and thyroxine-binding globulins exhibit a marked decrease in hormone affinity following cleavage and transition to the relaxed conformation (*21*, *30*). In CBG, this has been interpreted as a regulatory mechanism for corticosteroid release at sites of inflammation, where exposure to target proteases facilitates hormone release (*21*). By contrast, TpBBS appears unaffected by cleavage, suggesting that protease recognition is unlikely to mediate biliverdin release. This stability implies that maintaining biliverdin binding—and the associated spectral tuning—is critical for preserving the coloration and camouflage of *T. pulverata*. A high concentration of cleaved BBSs in normal physiological conditions in a glassfrog offers many avenues to investigate the function and stability of serpins in vivo and to explore the mechanism that permits the retention of optical properties even at high temperatures.

### Other roles of BBSs

The use of biliverdin and other tetrapyrroles as organismal coloration has been associated with thermoregulatory benefits in animals (*65*, *66*). For instance, unlike melanin-based coloration, which strongly absorbs visible and NIR light, and converts it into heat (*67*), biliverdin-based coloration in bird eggshells primarily absorbs UV and red wavelengths, with minimal absorption in the green and NIR portions of the spectrum (*65*, *68*). Because nonabsorbed light is scattered by the mineral matrix of the shell, eggs can reduce heat gain while still maintaining coloration for crypsis or other functions (*65*, *69*).

A similar principle may operate in frogs, where optical and thermal outcomes can arise from a coupled system involving pigment absorption, tissue-level scattering, and deeper structural reflectors (*4*, *36*). Light not absorbed by BBSs can be strongly reflected by broadband-reflecting tissues, such as mirrored peritonea and fascia (*4*), or transmitted through the body (*7*). Together, these processes shape both the external green phenotype and the internal distribution of incident radiation and may influence the fraction of light ultimately converted into heat relative to melanin-bearing species. The effectiveness of this mechanism is likely to depend on the concentration and spatial distribution of BBSs, as well as on co-occurring physiological and behavioral thermoregulatory strategies. In addition, thermal effects are expected to be strongly influenced by the spectral characteristics of the natural light environments inhabited by different species, with potentially larger effects in chlorotic frogs exposed to higher levels of solar irradiance (*43*). Like chlorophyll-bearing vegetation, BBS-containing frogs combine strong absorption of red wavelengths with substantial reflection and transmission of NIR light, which may reduce the fraction of incident solar radiation converted into heat. If these optical properties result in body temperatures that more closely match those of surrounding leaves, then they could also contribute to thermal camouflage by reducing thermal contrast with the background and potentially limiting detection by heat-sensing predators (*70*, *71*).

However, the heterogeneous distribution of BBS-containing lymph also raises the possibility of an alternative thermoregulatory role. Rather than reducing whole-body heat gain, localized accumulations of BBSs in plasma, lymphatic sacs, muscle, or other deep tissues could instead influence spatial patterns of light absorption and promote localized increases in temperature. Such localized hyperthermia can mitigate bacterial infections in other organisms (*72*), and basking behavior in amphibians—or experimental temperature increases—has similarly been associated with enhanced immune responses through elevated body temperature (*73*–*75*). Whether frogs can dynamically redistribute BBSs across tissues in ways that facilitate localized thermal effects remains unknown, but further theoretical and experimental work may help clarify potential links between BBS-based optical traits, thermoregulation, and immune function.

Alternatively, in this work, we documented that BBSs from treefrogs and the glassfrogs emit fluorescence in the NIR region of the spectrum when excited in the near-UV/violet or the far-red portions of the spectrum. While other amphibian-derived fluorophores in chromatophore cells are likely quenched in vivo (*36*), BBS fluorescence can be detected from multiple tissues, deep within the organism, such as the vasculature, muscles, lymph, and bones. Using fluorescence spectroscopy and Monte Carlo optical modeling, we demonstrated that the emission spectrum of BBSs in vivo matches that of the purified BBS, which suggests that under physiological conditions, BBSs circulating in the plasma and lymph preserve the same optical traits as when pure.

Whether fluorescence emission in the NIR plays a physiological role or is only a by-product of biliverdin binding to the serpin is unknown. A visual function for NIR emission would require visual systems with photoreceptors sufficiently sensitive to that portion of the spectrum, a condition not documented for purely Opsin-based detectors in nature and one that has been suggested to be physically unlikely (*76*). However, some organisms, like the deep-sea dragonfish, can use photosensitizers—usually chlorophyll derivatives—to extend their visual sensitivity to the far-red (*77*). This phenomenon has also been induced in living rod cells extracted from the axolotl *Ambystoma tigrinum* (*78*) and even in mouse models (*79*). Now, there is no information on the visual sensitivity of the frogs studied in this work or evidence suggesting that either the frogs or other interacting species (such as predators, parasites, etc.) might broaden their sensitivity using additional photosensitizers.

A potential visual function in communication requires an increase in contrast with the background that can be perceived by a visual system. Conversely, the leaf-like color produced by BBSs causes a reduction in the contrast, and since the emission of BBSs also overlaps with that of chlorophyll, it is likely that if there is a visual function of fluorescence emission, it would be more related to camouflage. More research is needed to thoroughly evaluate the physiological, ecological, and behavioral effects of fluorescence emission, including other possible nonvisual roles.

### Opportunities for bioinspiration

BBSs represent a notable example of functional diversification within the Serpin superfamily. Their stability, high affinity for biliverdin, and distinctive optical properties generate clear phenotypic effects that can be captured using fast, noninvasive spectroscopic and imaging techniques. Together, these features make the evolution of BBSs a powerful model for investigating fundamental questions in evolutionary biochemistry and serpin evolution, as well as the biochemical mechanisms that tune the colors of tetrapyrroles. In addition to their evolutionary relevance, the high affinity and stability of BBSs, combined with their fluorescence in the NIR—the optical transparency window—offer opportunities to explore their use as probes for in vivo imaging, particularly in contexts where the use of conventional visible-light fluorophores are limited due to the autofluorescence (*80*). Their strong biliverdin binding can help to circumvent challenges faced by other fluorophores that require endogenous biliverdin in mammals, where biliverdin is rapidly converted to bilirubin (*8*, *10*). Beyond fluorescence, the successful use of PAT to image BBSs in vivo demonstrates that their PA spectrum closely matches their absorption spectrum and that this property is preserved in living systems. This finding suggests that arboreal frog-derived BBSs could be used as a reliable contrast agent for deep-tissue PA imaging, especially for lymphatic applications where current nanoparticle-based optical contrast agents are often considered unstable and toxic (*81*, *82*). Expanding the characterization of BBSs across multiple frog lineages will not only provide new evolutionary insights but also pave the way for harnessing naturally occurring NIR fluorescence for biological imaging.

## MATERIALS AND METHODS

### Ethics declaration

All research procedures and colony care procedures were approved by the Institutional Animal Care and Use Committee of Duke University [protocol numbers A210-18-09 (#78458) and A174-21-08] and California Institute of Technology Protocol IA25-1913.

### Animal species and endogenous BBS purification

BBSs from adult glassfrogs *T. pulverata* and the adult treefrogs *B. punctata* and *S. lacteus* were extracted and purified as described (*4*).

### Size exclusion chromatography

A total of 5 μM BBS in 100 mM sodium phosphate buffer (pH 7.2) was applied to a preequilibrated Enrich size exclusion chromatography (SEC) 650 column (Bio-Rad) at 0.5 ml/min. The protein elution was detected at 280 nm. A SEC calibration curve was prepared with a set of globular protein standards ranging from 1.35 to 670 kDa (Gel Filtration Standard, Bio-Rad).

### Protein deglycosylation and crosslinking

Endogenous BBSs were deglycosylated using a Remove-iT PNGase F kit (NEB) with slight modifications from the manufacturer’s instructions. Briefly, 0.5 μl of 10× dithiothreitol (DTT) was added to a 5 μl of BBS solution (20 μg) in 1× PBS (pH 7.4) and incubated for 10 min at 55°C to partially denature the protein. One microliter of 10× Glycobuffer 2 and 2 μl of Remove-iT PNGase F were added, and the samples were incubated at 37°C overnight. Control conditions with PNGase F–free BBS solutions with 2 μl of high-performance liquid chromatography (HPLC)–grade water were incubated at the same temperature conditions to rule out temperature degradation effects. Protein crosslinking was performed using the BS3-d_0_/d_4_ crosslinking agent according to the manufacturer’s protocol.

### De novo transcriptome assembly

One specimen of *T. pulverata* was euthanized, and the liver was immediately excised and preserved in 1.5 ml of RNAlater (Thermo Fisher Scientific). Tissue was homogenized using Zirconium beads, and total RNA was extracted using the Quick-RNA Miniprep Kit (Zymo Research) following the manufacturer’s instructions. The extracted RNA was sent to Novogene for sequencing, using 150-bp pair-end reads. The raw sequences were cleaned using Trimmomatic v0.39 (*83*) run in pair-end mode (3′ end quality = 20; sliding window mean quality = 20, and minimum length = 36). Cleaned sequences were assembled using Trinity v2.15.2 (*84*).

### Mass spectrometry and protein sequencing

BBSs were subjected to SDS-PAGE and stained with Coomassie brilliant blue (CBB R250). The CBB-stained gel bands were cut out, destained, reduced with DTT, and alkylated with iodoacetamide, followed by digestion with Trypsin Gold (Promega). The trypsinized peptides were lyophilized and reconstituted in 20 μl of 60% acetonitrile/0.1% trifluoroacetic acid (TFA). Two microliters of each sample was analyzed using a 30-min gradient by nanoLC-MS/MS on an Orbitrap Fusion Lumos (Thermo Fisher Scientific). MS/MS was acquired in a high-resolution, data-dependent mode on an Orbitrap. Mass spectrometry data were analyzed using the software PEAKS (*85*). The identified peptides were matched against the protein database generated from the de novo assembly of the transcriptome of *T. pulverata.* To identify the site of cleavage of the C-terminal region of the endogenous TpBBS, native TpBBS was analyzed in an LTQ Orbitrap XL Hybrid Ion Trap-Orbitrap Mass Spectrometer (Thermo Fisher Scientific) paired with a Waters Acquity UPLC. MS data obtained from the Orbitrap were manually analyzed.

### Matrix-assisted laser desorption/ionization–time-of-flight

Molecular weight of purified BBSs was determined by MALDI-TOF on an Autoflex TOF/TOF mass spectrometer (Bruker). The MALDI matrix was prepared by combining 50 mg of sinapinic acid, 1 ml of HPLC-grade water, and 1 ml of acetonitrile, with 0.1% TFA. Samples were prepared mixing 1 μl of protein with 1 μl of matrix and cocrystalized at room temperature. For the molecular mass determination, a mixture consisting of four standard proteins—insulin, ubiquitin I, cytochrome C, myoglobin (Protein Calibration Standard I, Bruker)—ranging from 4 to 20 kDa was used.

### Sequence alignment and structure prediction

All protein sequences were aligned using T-Coffee (*86*). The resulting multiple sequence alignment was manually refined using Jalview (*87*). For estimating sequence identity and similarity, the N-terminal region of the serpins was not considered. The sequence alignment was visualized with Sequence Manipulation Suite (Color Align Properties) (*88*). Three-dimensional (3D) structure of the BBS was predicted using AlphaFold3 (*89*).

### Spectroscopy measurements

The absorbance spectrum of the protein and ligand was collected from 250 to 800 nm in a Jasco V-750 UV-Vis spectrophotometer. The final spectrum is background corrected with the appropriate buffer. BBS (1 to 2 μM) emission fluorescence was collected by exciting the samples at 390 or 630 nm and monitoring the emission from 650 to 750 nm in an Edinburgh FS5 fluorimeter, with excitation and emission bandwidths of 5 nm. The presented fluorescence spectrum is averaged over three accumulations.

The relative quantum yield of BBSs was estimated by measuring the integrated fluorescence intensity and absorbance of the sample and reference fluorophores. The quantum yield was derived directly from the ratio of their linear regression slopes. The fluorescence emission of BBSs and Cy5.5 (absorbance at 650 nm ranging from 0.01 to 0.1) was measured from 670 to 900 nm by exciting the samples at 650 nm. Cy5.5 (QY = 0.28) dissolved in PBS was used as the standard, as its excitation and emission spectra overlap with those of BBSs.

### Thermal unfolding

Thermal unfolding of BBSs was monitored by measuring the absorbance at 376 and 390 nm. BBSs in 1× PBS (*A*_390_ = 0.16) were gradually heated from 22° to 95°C at a rate of 1°C/min, and the absorbance changes (A390/A376) were monitored in an Agilent Cary UV-Vis spectrophotometer or an Edinburgh FS5 spectrometer connected to the Peltier controller. The melting point was calculated from the first derivative of the absorbance signal as a function of time (dA/dT), and the peak of the derivative corresponds to the melting temperature of the protein.

### Cloning, protein overexpression, and purification

The coding sequence corresponding to the TpBBS, BpBBS, and SlBBS was synthesized from GeneArt. BBS genes were subcloned into pBG106 expression vector (Center for Structural Biology, Vanderbilt University) through In-fusion HD Cloning (Takara Bio Inc.). TpBBS and SlBBS were overexpressed in *E. coli* Rosetta2(DE3) cells. The transformed bacterial cells were cultured at 37°C to an optical density at 600 nm (OD_600_) of 0.6, and then the overexpression was induced with 0.2 mM isopropyl-β-d-thiogalactopyranoside (IPTG) for 16 hours at 22°C with constant shaking. BpBBS was overexpressed in *E. coli* Rosetta2 (DE3) cells grown to OD_600_ = 1.0 and then induced with 1 mM IPTG for 16 hours at 16°C with constant shaking. Cells were pelleted and resuspended to homogeneity in lysis buffer [20 mM tris (pH 8), 150 mM NaCl, and two protease inhibitor tablets (Roche)]. The cells were lysed by sonication, and the cell lysate was clarified by centrifuging at 10,000*g* for 40 min. The lysate was loaded onto the Nickel Sepharose 6 Fast Flow affinity column (Cytiva) preequilibrated with the lysis buffer. For SlBBS and TpBBS, the nonspecific impurities were washed with lysis buffer containing 50 mM imidazole, and the bound protein was eluted with lysis buffer containing 300 mM imidazole. The eluted protein was dialyzed against QA buffer [20 mM tris (pH 8), 25 mM NaCl, and 1 mM DTT] overnight at 4°C. The dialyzed protein was loaded onto an Uno Q6 (Bio-Rad) column connected to Bio-Rad NGC FPLC and eluted with a salt gradient ranging from 25 to 500 mM NaCl. The peak fractions containing BBS were pooled together, dialyzed against storage buffer [20 mM tris (pH 8), 50 mM NaCl, and 10% glycerol] overnight. The protein was then concentrated, aliquoted into small fractions, and flash frozen. For BpBBS, nonspecific proteins were extensively washed with 20 mM imidazole, and BpBBS was eluted with 300 mM imidazole; with no further purification, the eluate was buffer-exchanged into 1× PBS.

### Relaxed TpBBS preparation

The endogenous TpBBS was identified in the relaxed confirmation with an elastase recognition site in the RCL. The relaxed TpBBS was prepared by incubating the rTpBBS with human neutrophil elastase (Innovative Research Inc.) at a 1:3 ratio at 25°C for 6 hours in 1× PBS.

### Determination of biliverdin, apo-BBS, and holo-BBS concentrations

Apo-BBS concentrations were determined by measuring absorbance at 280 nm (*90*) (ε_280,TpBBS_ = 33,350 M^−1^ cm^−1^, ε_280,BpBBS_ = 28,880 M^−1^ cm^−1^, ε_280,SlBBS_ = 35,870 M^−1^ cm^−1^). For the quantification of free biliverdin (Cayman Chemical) in 1× PBS, we determined the extinction coefficient at the Soret band (ε_376nm_ = 39,900 M^−1^ cm^−1^) and Q band (ε_672nm_ of 11,200 M^−1^ cm^−1^) (fig. S3). A known mass (10 mg, 95% purity) of biliverdin was dissolved in dimethyl sulfoxide at 9.5 mg/ml and further diluted to different concentrations in 1× PBS, and the absorbance spectrum was recorded from 250 to 850 nm. The molar extinction coefficient of biliverdin in 1× PBS was determined from the slope of the absorbance versus concentration plot (fig. S3). The extinction coefficients of biliverdin bound to the protein at the Soret and Q bands were determined using recombinant BBSs. A fixed concentration of biliverdin was titrated with increasing concentrations of apo-BBS until no further changes in absorption were observed.

### Ligand binding and determination of dissociation constants (*K*_d_)

Dilutions of recombinant BBS were prepared in a flat black 96-well plate (Corning) spanning 0.2 to 30 nM in 1× PBS, and the *K*_d_ was captured within the binding regime (*27*). A total of 15 nM biliverdin was added to each well, and fluorescence emission was collected at 697 nm (rTpBBS), 710 nm (rBpBBS), and 707 nm (rSlBBS) with 20-nm bandwidths for excitation and emission wavelength at an optimal gain. Fluorescence intensities were measured using a Tecan Spark plate reader with a small humidity cassette filled with ultrapure water to reduce evaporative sample loss, and scans were taken once every hour for 24 hours at room temperature. The equilibrium dose-response curve was plotted (normalized for relative fluorescence intensity versus concentration), and the *K*_d_ value was determined by fitting the data with the Sigmoidal curve equation on GraphPad Prism version 10.6.1 for Windows, GraphPad Software, Boston, Massachusetts USA, www.graphpad.com.

### In vivo spectroscopy

We measured the reflectance spectra of the frogs as previously described (*7*). Light from the dorsum of the frog was measured using an integrating sphere (Ocean Optics ISP-REF; port diameter, 10.32 mm) connected to a multichannel spectroradiometer (Ocean Optics, USB 2000). We used the built-in tungsten halogen lamp within the Integrating sphere ISP-REF for the light source. To obtain reflectance measurements, a Spectralon diffuse 100% reflectance standard was used (Labsphere Inc.) to calibrate the device before each spectrum acquisition.

We measured the reflectance spectra of leaves used as background substrates using the same integrating sphere and spectroradiometer setup described above. We recorded spectra from the abaxial and adaxial surface of leaves of floating fern (*Salvinia biloba*), water lettuce (*Pistia stratiotes*), heartleaf philodendron (*Philodendron hederaceum*), and water primrose (*Ludwigia peploides*). Before each acquisition, we calibrated the spectroradiometer using a Spectralon diffuse 100% reflectance standard (Labsphere Inc.) and recorded the reflectance spectra relative to this standard under identical illumination conditions.

The in vivo fluorescence emission was measured in adult specimens of *T. pulverata.* To simulate the effect of BBS absorption on the frog’s emission spectra, we performed a 2D Monte Carlo simulation using a three-layered system consisting of a broad-band reflector, a subcutaneous lymph layer with varying BBS concentrations, and an outer skin layer. A single fluorescent point source was defined for the simulation in the BBS-filled medium. The simulation was performed using the MCXLAB simulation package, which is the native MEX version of MCX for MATLAB (*91*, *92*). The simulation grid comprised a 300 × 300 matrix of isotropic pixels with a pixel width of ∼3.3 μm, corresponding to a total grid area of 1 mm^2^. The medium was split into three layers; an open-air background layer, a BBS-containing layer (split into skin and lymph), and an ideal specular reflector layer. The BBS skin and lymph layers were each 0.1 mm thick, with the reflector layer below it also being 0.1 mm thick. Four optical properties were defined for each layer in the model: absorption coefficient, scattering coefficient, scattering anisotropy (*g*), and refractive index. The scattering and absorption coefficients for the BBS containing layers were based on the empirically measured absorption and scattering spectra of BBS (see below). The scattering anisotropy and refractive index were defined as 0.9 and 1.4 for each layer in the model, respectively. One hundred seventy-one simulations were performed independently and subsequently, to model emission using the optical properties of BBS from 650 to 820 nm in increments of 1 nm. For each simulation, a single fluorescent-point-source pulse emitted light from the center of the BBS-filled layer, and the cumulative light reaching a point detector defined in the open-air layer was calculated. The intensity of the initial point source of light was defined using the empirical emission spectrum of BBS. Fifty million photon packets were modeled for each simulation, with a temporal resolution of 0.1 ps for a duration of 5 ps. Following the 171 simulations, the resulting relative emission profile was plotted on the basis of the total light reaching the detector for each of the wavelengths simulated.

To estimate the skin’s optical properties, we applied an inverse adding-doubling approach to reflectance and transmittance spectra acquired using an integrating sphere with a 10-mm port and a 1-mm incident light beam on the skin surface (*93*). In all the simulations, we assumed that the asymmetry parameter is 0.9 for the skin.

To more clearly show the spectral features of BBSs in the optical properties of the frogs, we estimated the frogs’ remission spectra using a Kubelka-Munk approach applied to the measured reflectance data (*94*). While this approach has limitations—particularly the assumptions of Kubelka-Munk theory regarding the requirement of an infinitely thick layer, isotropic scattering behavior, and constant scattering—it provides a useful approximation to qualitatively resolve spectral features in the frog spectra. Because the Kubelka-Munk remission function is proportional to chromophore concentration in turbid media, it facilitates comparison between BBS absorbance and tissue optical properties. Carotenoid contributions to the reflectance spectra, particularly in the 400- to 500-nm absorption region, were investigated by performing a chloroform extraction of the dorsal skin from an adult *T. pulverata*.

### NIR fluorescence imaging

Fluorescent images were acquired using an NIR high-sensitivity InGaAs camera with a spectral range of 600 nm to 1.7 μm (Ninox 640 II, Raptor Photonics). Excitation was provided either by an LED source with a 660-nm peak wavelength and a 20-nm full width at half maximum (FWHM) (Thorlabs, M660L4) or a 635-nm diode laser (Civil Laser Industries, LSR635CP-6 W) with an FWHM of <2 nm. Emission was detected using band-pass filters [Thorlabs 700 ± 10 nm (FBH700-10), 750 ± 10 nm (FBH750-10), and 800 ± 10 nm (FBH800-10)] and long-pass filters [1050 nm (FELH051050) and 1200 nm (FELH051200) cut-on].

### 3D PA and US imaging

For multispectral wide-field imaging of the frogs, we used a custom-built linear array–based 3D PAT system based on diffractive acoustic tomography (*95*). The system allowed for isotropic 3D PA and ultrasound (US) imaging of whole frogs. The system used a linear array transducer (L7-4, Philips) combined with an optically transparent single slit to enhance elevational resolution. Acoustic data from the transducer were acquired by a 256-channel data acquisition system (Vantage 256, Verasonics Inc.). For PA imaging, an Nd:YAG laser generated pulses (10-ns pulse width and 10-Hz pulse repetition rate) that were delivered to the frog through a dual-branch line fiber bundle (Dolan Jenner) from the ventral side. Before coupling into the line fiber bundle, a beam sampler directed <5% of the laser pulse energy into a power meter (Ophir) later used for energy normalization. The Nd:YAG laser pumped an optical parametric oscillator (Continuum Inc.) to allow for tunable wavelength control from 680 to 1000 nm. Laser pulsing and ultrasonic detection are controlled by an embedded device with FPGA module (myRIO-1900, NI). Each volumetric dataset for each wavelength used to image the frogs required ∼30 s, with 300 scanning steps (step size, 0.1 mm) defining a volume of 4 cm × 3 cm × 2 cm, covering the entire frog, with an isotropic spatial resolution of ∼400 μm. To evaluate if BBS absorption in vivo matches the spectral distribution of purified BBS, we injected purified TpBBS into optically and acoustically transparent microdialysis tubes and obtained the PA spectra for comparison.
