# Supplementary material for: Serpin-driven green camouflage and NIR fluorescence in frogs
Source: Sci Adv. 2026 Sep 16;12(38):eaeg5510. doi: 10.1126/sciadv.aeg5510 (PMC13580550; doi:10.1126/sciadv.aeg5510)
Supplement: Supplementary file 1 [file sciadv.aeg5510_sm.pdf]

Science Advances
AAAS

Supplementary Materials for

Serpin-driven green camouflage and NIR fluorescence in frogs

Ravichandran Vignesh *et al.*

Corresponding author: Junjie Yao, junjie.yao@duke.edu; Carlos Taboada, ctaboada@caltech.edu

Sci. Adv. **12**, eaeg5510 (2026)
DOI: 10.1126/sciadv.aeg5510

The PDF file includes:

Figs. S1 to S9
Legend for movie S1

Other Supplementary Material for this manuscript includes the following:

Movie S1

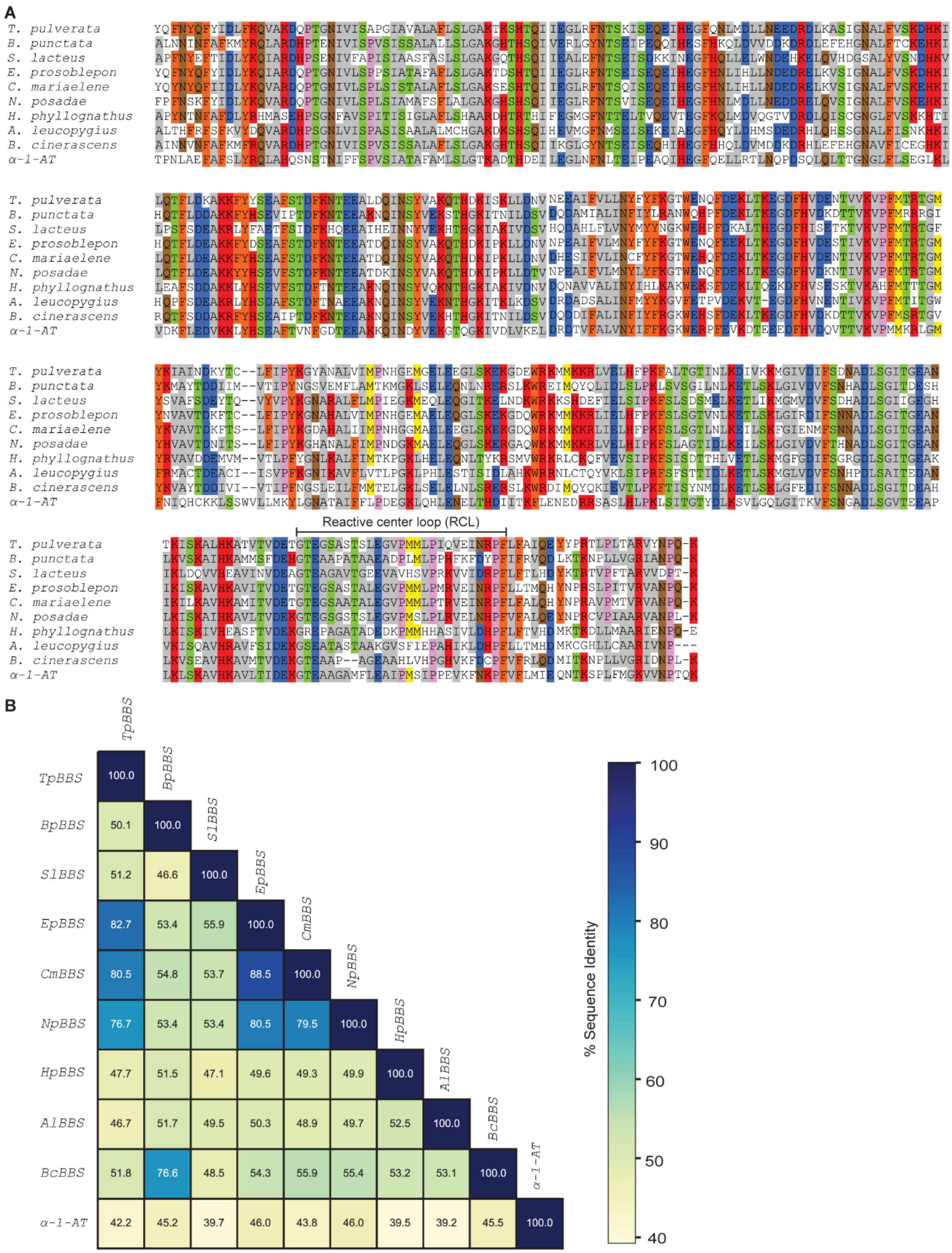

Fig. S1. Sequence Analysis. (A) Multiple sequence alignment of various BBSs from glassfrogs and treefrogs along with human alpha-1-antitrypsin. **(B)** Sequence identity matrix generated from the multiple sequence alignment.

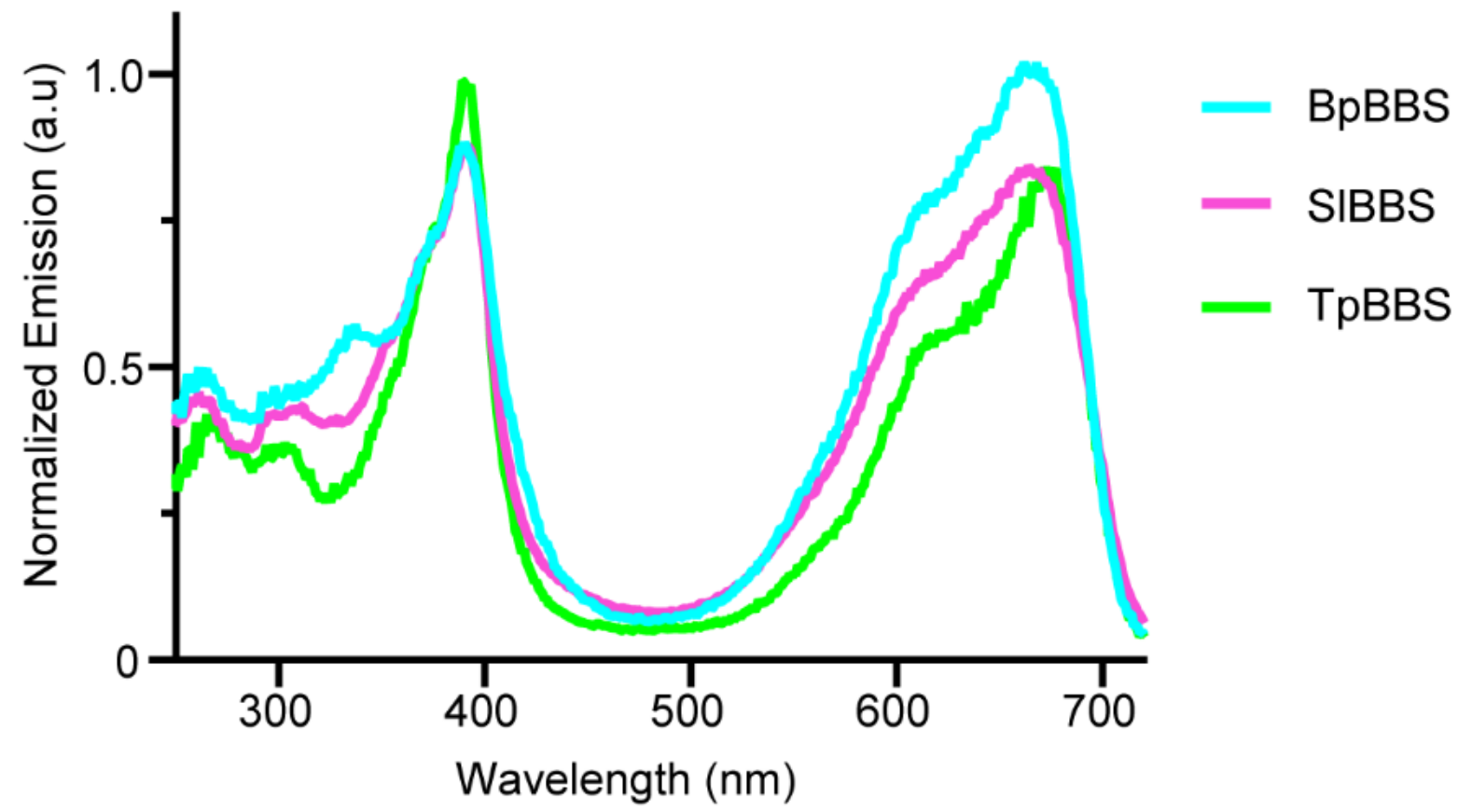

Fig. S2. Excitation spectra of BBS proteins. Excitation spectra of BpBBS, SlBBS, and TpBBS were collected from 250–720 nm while monitoring fluorescence emission at 740 nm. All three proteins exhibit a major excitation band near 390 nm, and a Q band characterized by a peak at 650-680 nm and a shoulder around 630 nm.

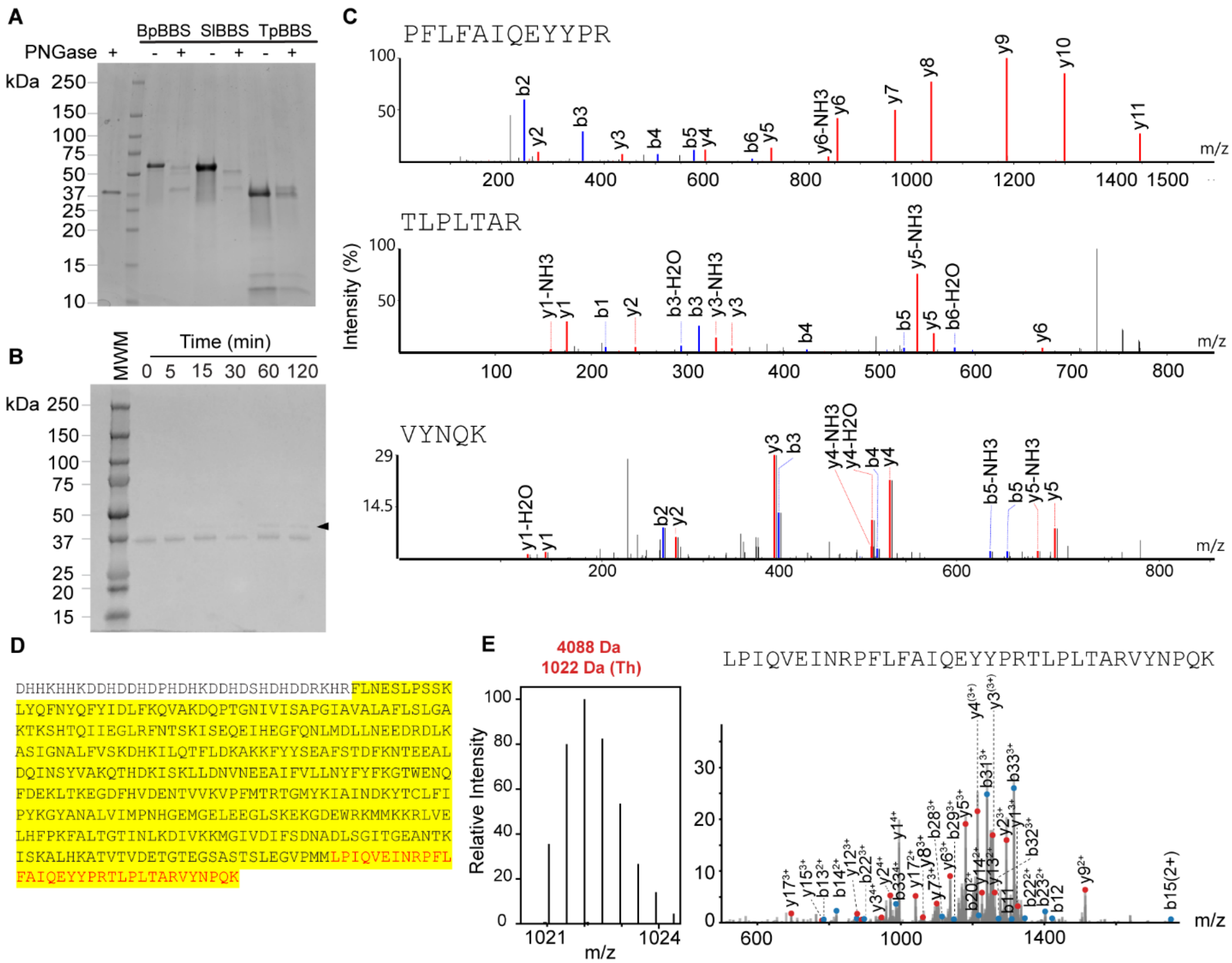

Fig. S3. Mass Spectrometry of BBS. (A) Glycosylation in BBS. SDS PAGE gel showing mobility differences between PNGase F-treated and untreated BBS. Treatment with PNGase F revealed two bands for BpBBS and one for SlBBS, consistent with the number of glycosylation sites inferred from their sequences. TpBBS did not exhibit any changes between the treated and untreated conditions. **(B)** BS3 d_0-d_4 crosslinking of endogenous TpBBS from 0-120 minutes incubation showed a band with a larger molecular weight (black arrow) – compatible with the addition of the C terminus – appearing after 15 minutes. **(C)** Bottom-up proteomics (in gel tryptic digestion) of the crosslinked band from the gel in (B). Analyses of LC-MS/MS revealed multiple fragments within the C-terminus of TpBBS. **(D)** The coverage of the bottom-up mass spectrometry analysis of TpBBS – highlighted in yellow – increases from 80% to 91% when the protein is crosslinked. C-terminus of TpBBS after the scissile bond is shown in red. **(E)** LC-MS/MS of endogenous TpBBS revealed a fragment of 4,088 Da aligning which aligns with the predicted molecular weight of the C-terminus of the protein. MS (left) and MS/MS of 1022 m/z (4+) peak (right).

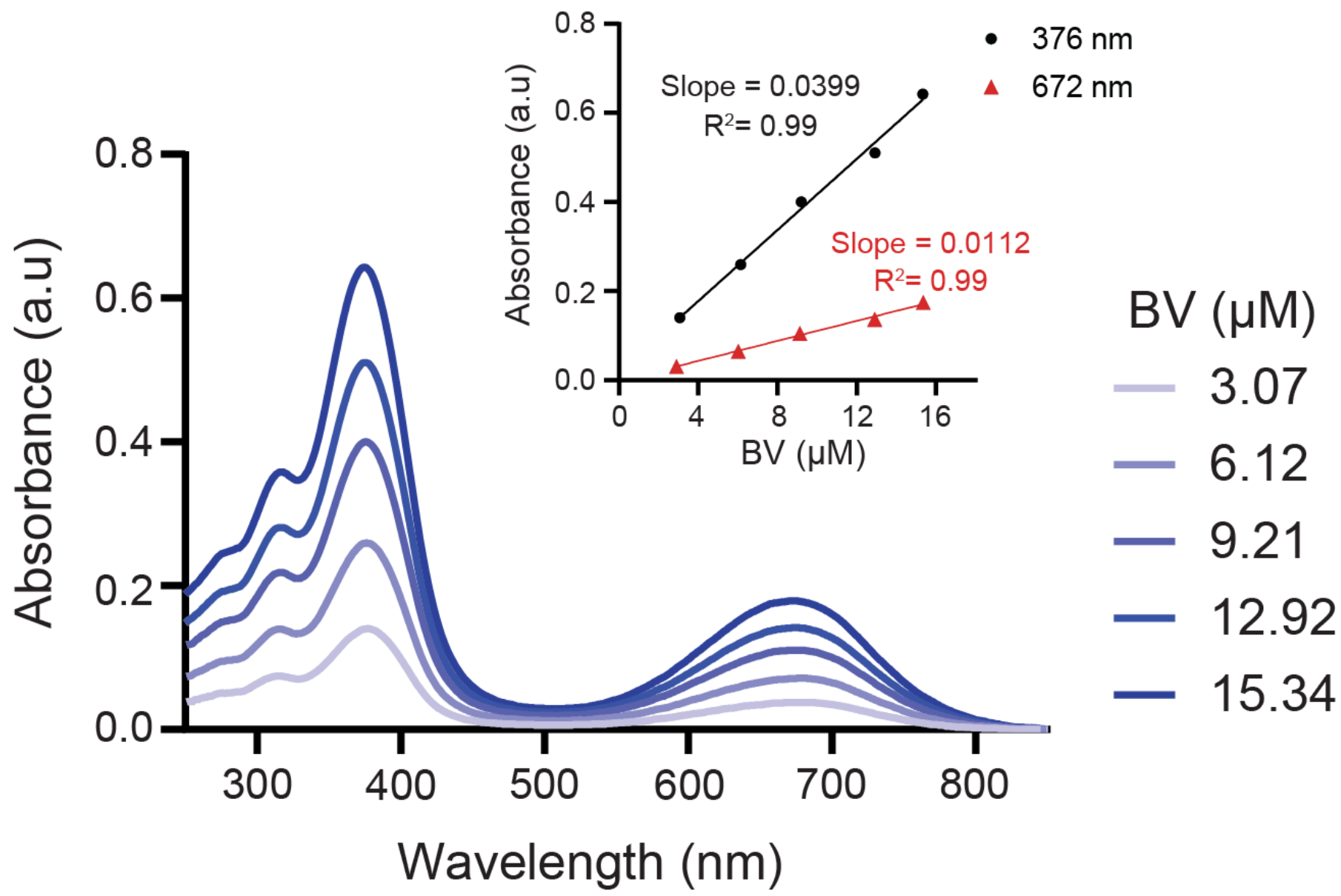

Fig. S4. Molar extinction coefficient of biliverdin in aqueous solution. Absorption spectrum of various concentrations of biliverdin in 1X PBS. *Inset*, Absorbance at 376 and 672 nm were plotted against the increasing concentrations of biliverdin. The slopes (molar absorption coefficients) were determined from the linear regression of the plotted values as $\varepsilon_{376\ nm} = 39{,}900\ M^{-1}cm^{-1}$ and $\varepsilon_{672\ nm} = 11{,}200\ M^{-1}cm^{-1}$.

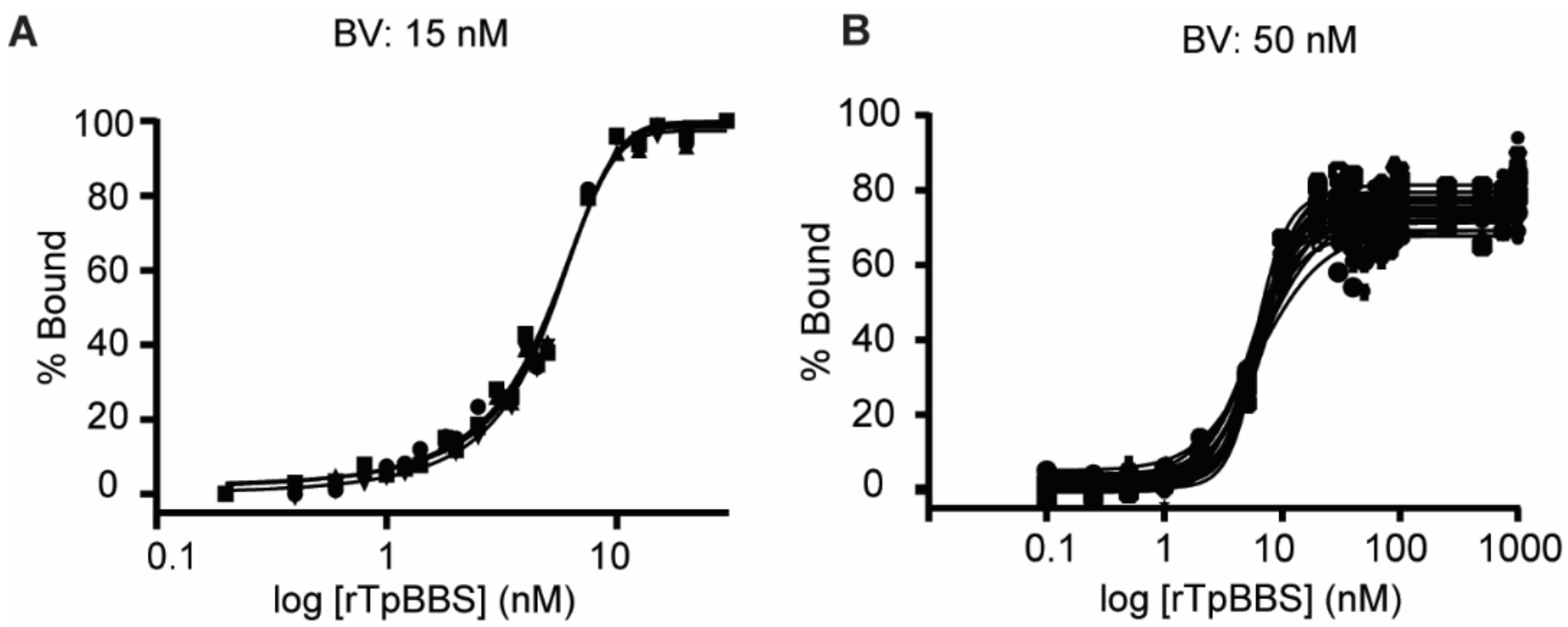

Fig. S5. Binding regime of rTpBBS. The fluorescence of biliverdin (BV) bound to rTpBBS remained stable over time, indicating that binding equilibrium was reached. This was observed in **(A)** with 15 nM BV over 0–4 h and **(B)** with 50 nM BV over 0–24 h. The average apparent K_d values calculated from these curves were 5 nM (A) and 6.2 nM (B). Further lowering the BV concentration reduced fluorescence below the detection limit.

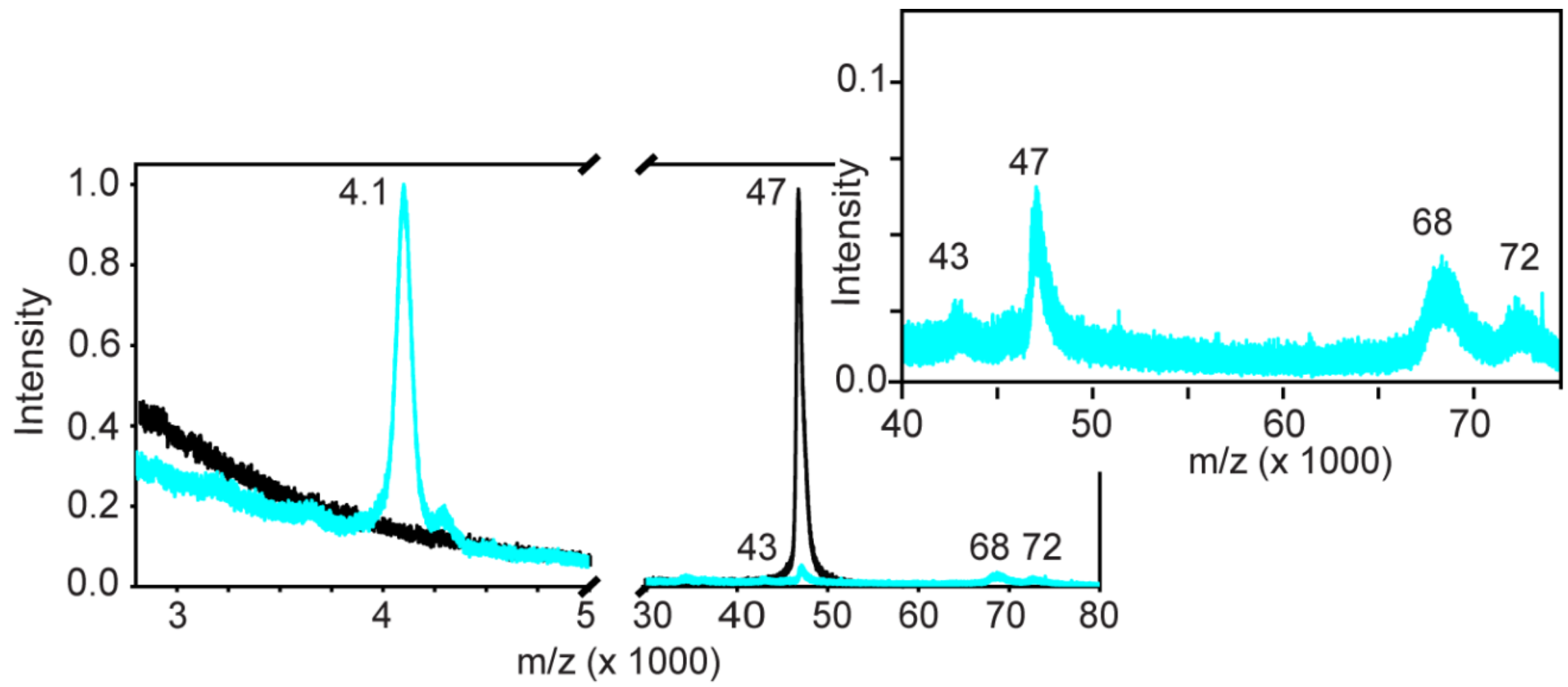

Fig. S6. MALDI-TOF of TpBBS in relaxed- (cyan) and stressed- (black) conformation. The appearance of the ~4.1 kDa signal indicates the cleavage of TpBBS by human neutrophil elastase, a fragment not observed in untreated TpBBS. The 43 kDa and 47 kDa peaks correspond to the cleaved (relaxed) and native (stressed) TpBBS. The 72 kDa and 68 kDa peaks correspond to elastase complexed with the cleaved TpBBS and native TpBBS, respectively. Elastase is ~26 kDa.

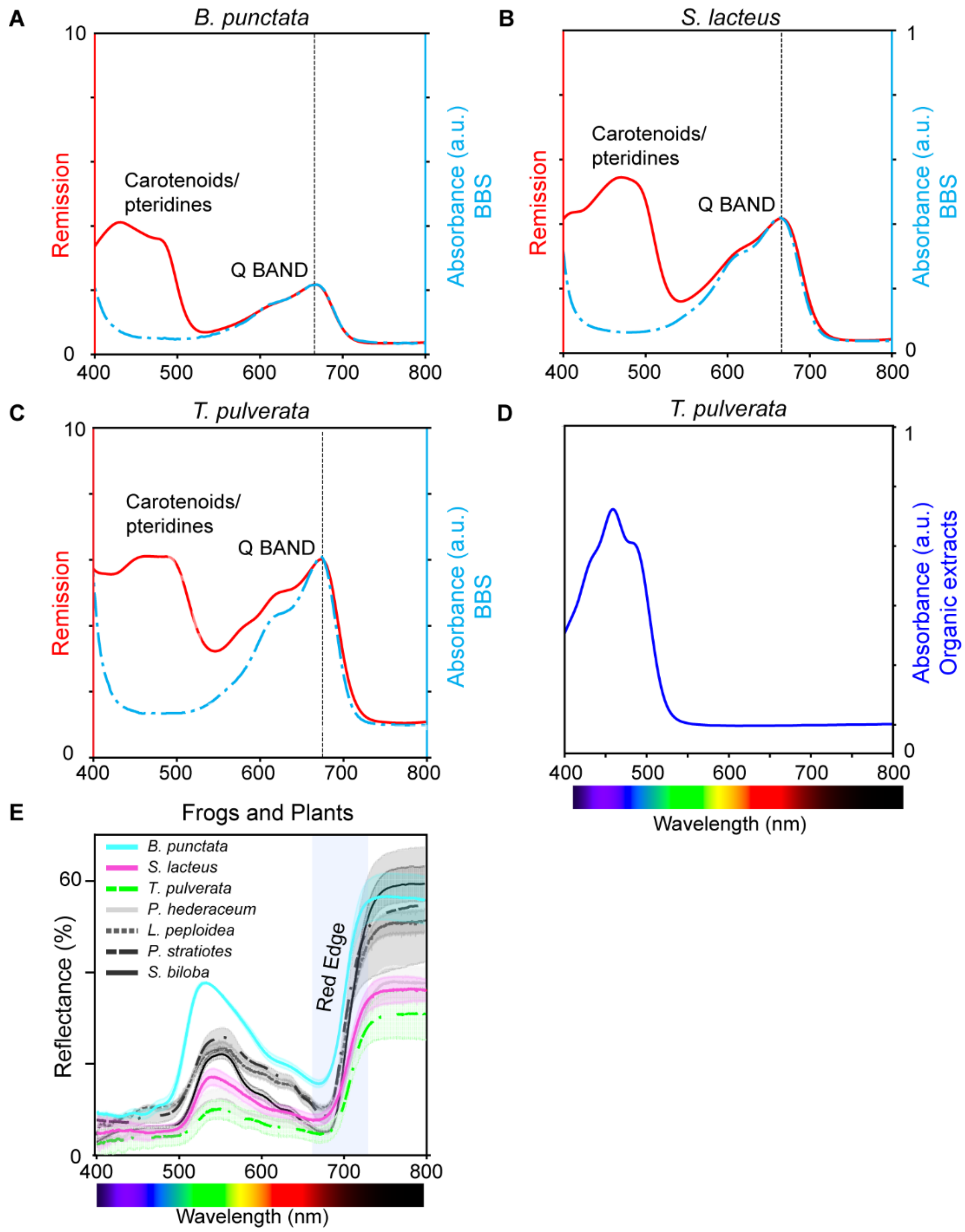

Fig. S7. Whole-body spectral properties in two treefrogs and a glassfrog. (A–C) Remission spectra derived from frog reflectance spectra (see Fig. 5). The absorbance spectra of BBSs closely match the remission spectrum in the red region. The peak between 400 and 490 nm is attributed to carotenoids present in frog skin. **(D)** Absorbance spectrum of chloroform extracts obtained from the skin of *T. pulverata*. The solubility and absorbance properties of these extracts are consistent with those of carotenoids. Furthermore, their absorbance maximum closely coincides with the region of highest remission and lowest reflectance (A–C and Fig. 5) and is consistent with previously reported transmission and attenuation spectra of isolated amphibian skin that reveal high absorbance by carotenoids in the blue portion of the spectrum. **(E)** Averaged reflectance spectra and standard deviation (shaded) of *in vivo* dorsal side of frogs (*B. punctata* n = 5, *S. lacteus* n = 7, *T. pulverata* n = 5, mean ± SD) compared to several species of plants. The red edge (shaded light blue) corresponds to a region of sharp increase in reflectance that is present in both the frogs and the plants, revealing a close correspondence in their spectral signatures.

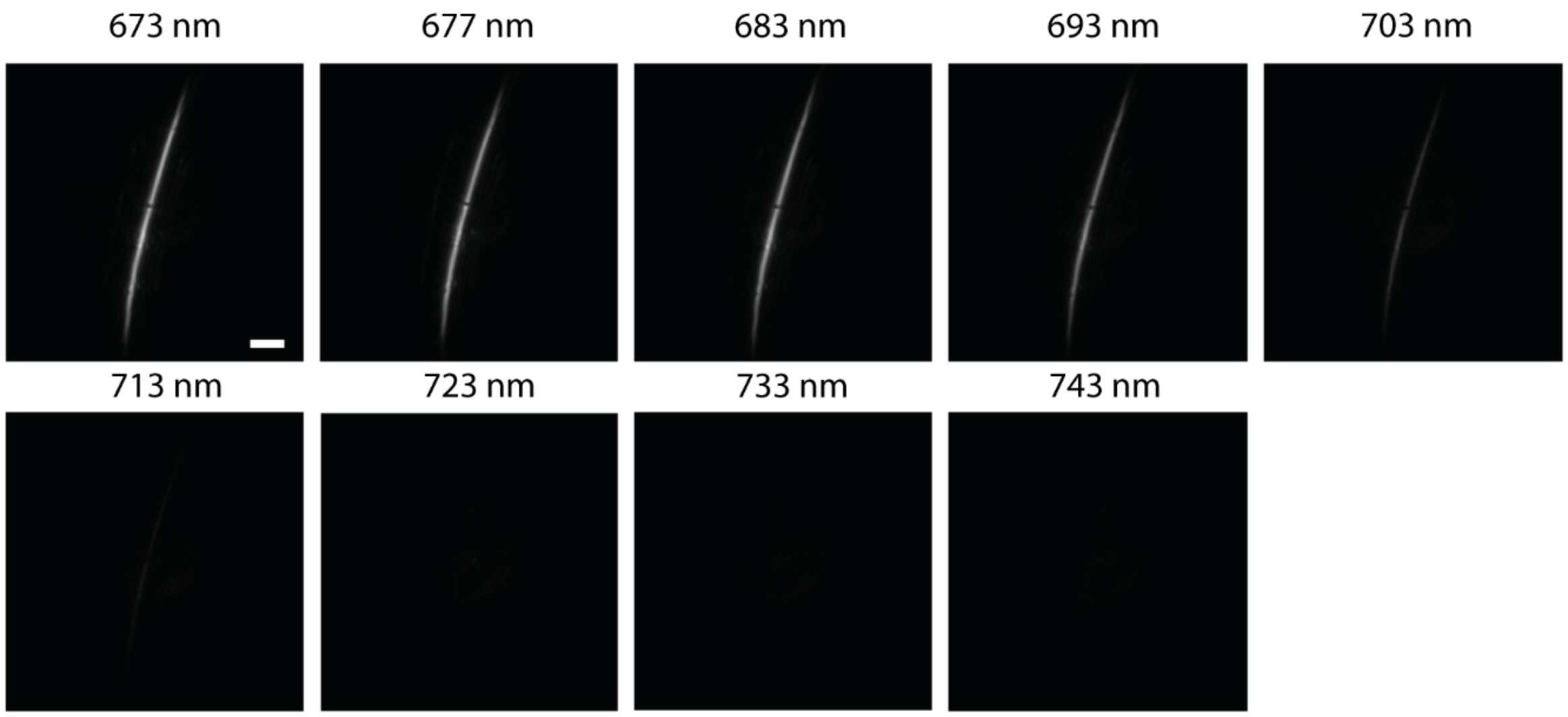

Fig. S8. PA images of BBS-filled tubes for spectral determination. TpBBS is filled into a silicone tube with an inner diameter of 0.31 mm and an outer diameter of 0.64 mm. Then, PA images are acquired at incremental wavelengths from 673 to 743 nm. The averaged PA amplitude of the tube at each wavelength is used for the determination of the PA spectra in **Fig. 6**. Scale bar: 5 mm.

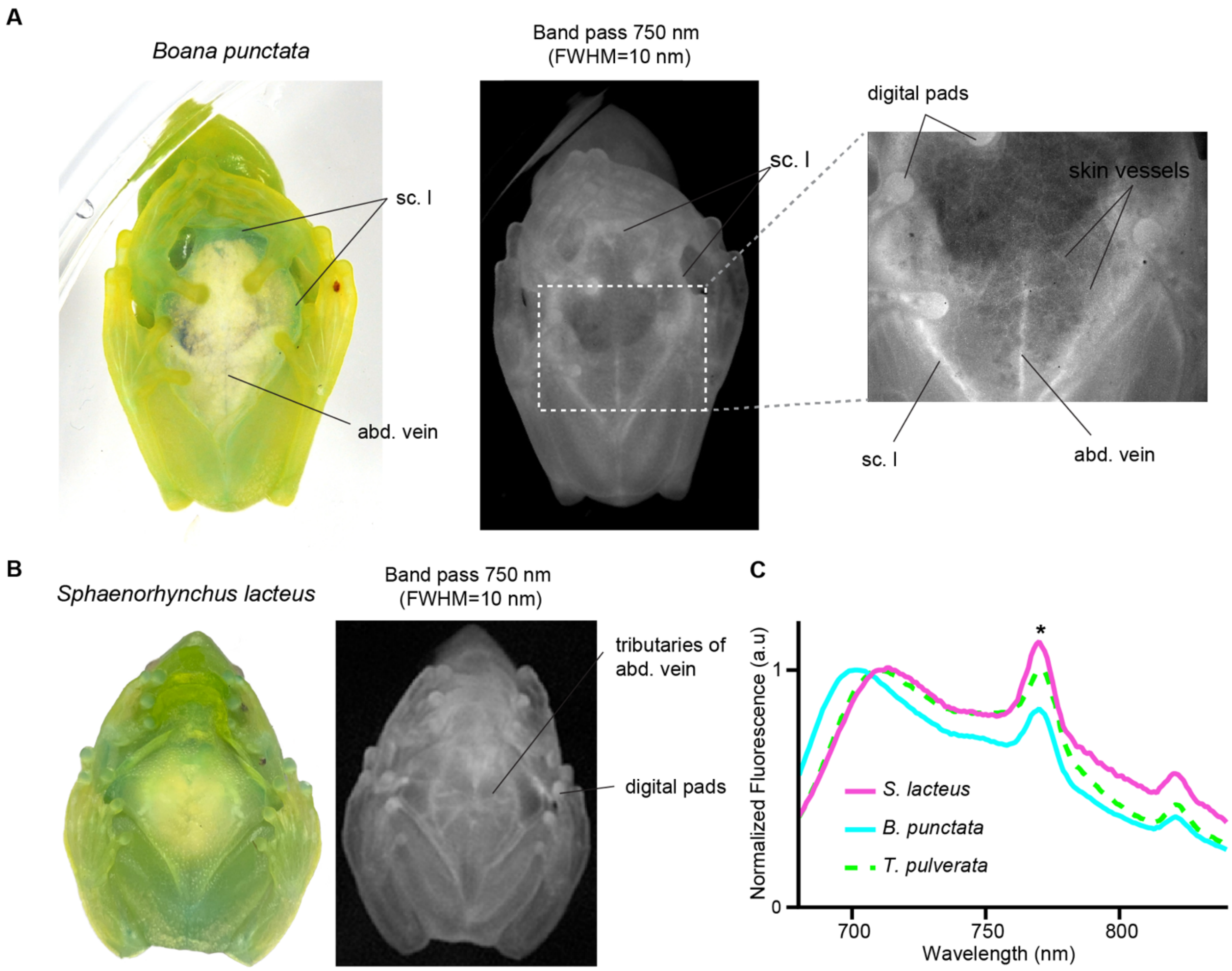

Fig. S9. BBS-mediated NIR fluorescence. Brightfield and fluorescence images of **(A)** *B. punctata* and **(B)** *S. lacteus,* used to visualize the internal tissues. *Inset,* enlarged portion of the ventral side of *B. punctata* highlights the anatomical structures**.** *abd. vein.*= abdominal vein; *sc. l.* = subcutaneous lymph. **(C)** Normalized *in vivo* emission spectra collected under 630 nm excitation. All three species exhibit near-infrared fluorescence with emission maxima near 690–720 nm. *- Raman peak.

Movie S1.

Ventral view of a glassfrog (*T. pulverata*) under white light illumination (left) and under 660 nm light to excite BBS fluorescence emission (750 nm bandpass filter, FWHM = 10 nm). Frog NIR fluorescence is shown in grayscale (center) or as orange pseudocolor (right). BBS is distributed in the vasculature, lymph, bones, and muscles.
